# Rapid whole-genome sequencing decreases infant morbidity and cost of hospitalization

**DOI:** 10.1038/s41525-018-0049-4

**Published:** 2018-04-04

**Authors:** Lauge Farnaes, Amber Hildreth, Nathaly M. Sweeney, Michelle M. Clark, Shimul Chowdhury, Shareef Nahas, Julie A. Cakici, Wendy Benson, Robert H. Kaplan, Richard Kronick, Matthew N. Bainbridge, Jennifer Friedman, Jeffrey J. Gold, Yan Ding, Narayanan Veeraraghavan, David Dimmock, Stephen F. Kingsmore

**Affiliations:** 1 Rady Children’s Institute for Genomic Medicine, San Diego, CA USA; 2 Department of Pediatrics, University of California San Diego, San Diego, CA USA; 3 Torrey Pines Health Group Inc., San Diego, CA USA; 4 Department of Family Medicine and Public Health, University of California San Diego, San Diego, CA USA; 5 Department of Neurosciences, University of California San Diego, San Diego, CA USA

## Abstract

Genetic disorders are a leading cause of morbidity and mortality in infants. Rapid whole-genome sequencing (rWGS) can diagnose genetic disorders in time to change acute medical or surgical management (clinical utility) and improve outcomes in acutely ill infants. We report a retrospective cohort study of acutely ill inpatient infants in a regional children’s hospital from July 2016–March 2017. Forty-two families received rWGS for etiologic diagnosis of genetic disorders. Probands also received standard genetic testing as clinically indicated. Primary end-points were rate of diagnosis, clinical utility, and healthcare utilization. The latter was modelled in six infants by comparing actual utilization with matched historical controls and/or counterfactual utilization had rWGS been performed at different time points. The diagnostic sensitivity of rWGS was 43% (eighteen of 42 infants) and 10% (four of 42 infants) for standard genetic tests (*P* = .0005). The rate of clinical utility of rWGS (31%, thirteen of 42 infants) was significantly greater than for standard genetic tests (2%, one of 42; *P* = .0015). Eleven (26%) infants with diagnostic rWGS avoided morbidity, one had a 43% reduction in likelihood of mortality, and one started palliative care. In six of the eleven infants, the changes in management reduced inpatient cost by $800,000–$2,000,000. These findings replicate a prior study of the clinical utility of rWGS in acutely ill inpatient infants, and demonstrate improved outcomes and net healthcare savings. rWGS merits consideration as a first tier test in this setting.

## Neonatology: Rapid sequencing of sick babies is useful and cost-effective

Rapid whole-genome testing for babies in intensive care yields improved health outcomes and lowers medical costs. Stephen Kingsmore from Rady Children’s Institute for Genomic Medicine in San Diego, California, USA, and colleagues retrospectively analyzed a cohort of 42 infants who had their genomes decoded in 2–5 days. This quick turnaround DNA sequencing identified disease-causing genetic defects in 18 infants, 13 of whom then had a change in medical or surgical treatment that helped alleviate symptoms or prevent death. As a result, many of those babies had shorter hospital stays and reduced healthcare costs. By comparison, standard genetic testing yielded a diagnosis in four cases, and prompted change of care for only one individual. The findings confirm Kingsmore’s results from another hospital, and suggest rapid sequencing should be more widely adopted for critically ill infants.

## Introduction

Genetic disorders and congenital anomalies affect ~6% of live births, and are the leading reason for hospitalization and mortality in infants.^1–4^ Of 14% of US newborns admitted to neonatal intensive care units (NICU), those with genetic disorders have longer hospitalizations and higher resource utilization.^1,5^ While early etiologic diagnosis in such infants enables optimal outcomes, it is exceptionally difficult to deliver for genetic diseases since they number over 8000 and presentations are often atypical from classical descriptions.^6^ Moreover, they represent the leading cause of NICU and paediatric intensive care unit (PICU) mortality, with most deaths following palliative care decisions. Family counselling regarding palliative care often is impeded by absence of etiologic diagnosis.^7^

Rapid whole-genome sequencing (rWGS) provides a faster diagnosis, enabling precision medicine interventions in time to decrease the morbidity and mortality of infants with genetic diseases.^6^ Furthermore, rWGS facilitates end-of-life care decisions that can alleviate suffering and aid the grieving process. However, published evidence demonstrating the effectiveness of rWGS in improving outcomes in infants is insufficient to endorse large-scale implementation;^8^ It is limited to case reports, and one retrospective study (Level III evidence).^9^ Examination of reproducibility is imperative. Here, we report such an examination.

## Results

**Fig. 1:**
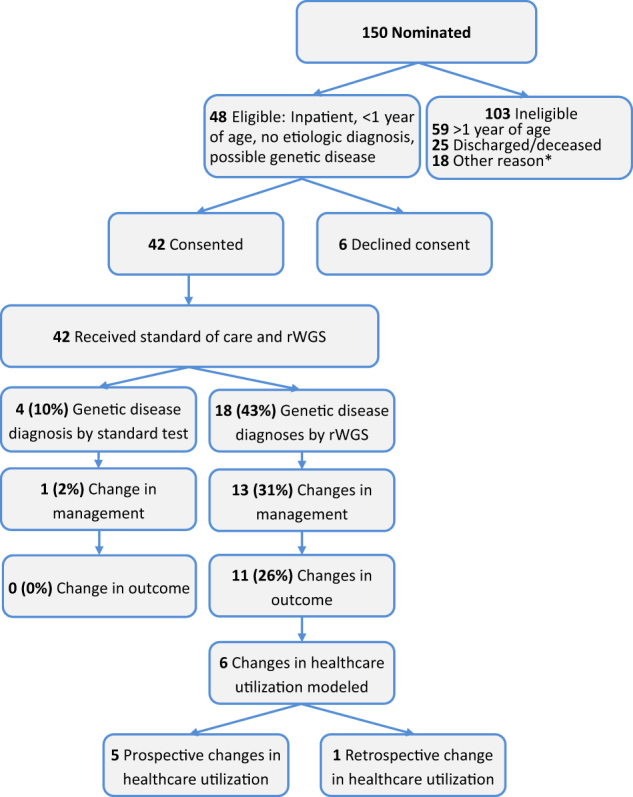
Flow diagram of the proportion of inpatient infants who were enrolled, received genetic disease diagnoses by rWGS or by standard tests, had consequent acute changes in management (precision medicine), resultant change in outcome, and analysis of impact on acute healthcare utilization. *Include: diagnosis obtained via clinical testing, symptoms determined to not likely be due to a genetic etiology, and/or parents unavailable for consent

Parents provided consent for 42 of 48 eligible infants (88%, Fig. 1). While the intent was trio rWGS (parents and affected infant), rWGS was performed on 29 trios and 1 quad (parents and two affected siblings), nine mother–infant duos, and three singletons.

Most probands were term births with normal weight (Table 1). Fifty nine percent were Hispanic/Latino. Consanguinity was uncommon (2%). The severity of illness was high: 71% were in a regional NICU, PICU or cardiovascular intensive care unit, 76% received respiratory support and 40% inotropic support. Disease phenotypes were highly diverse (Table 1, S1). Most presentations were complex, with several systems affected. The most common presentation was multiple congenital anomalies (29%).

**Table 1:** Demographic and clinical characteristics of the 42 proband, inpatient infants

			Total	Rapid WGS	
			(*n* = 42)	Diagnostic (*n* = 18)^a^	Negative (*n* = 24)
Sex	Female		21 (50%)	10 (56%)	11 (46%)
	Male		21 (50%)	8 (44%)	13 (54%)
Race and ethnicity	Caucasian		12 (29 %)	6 (33%)	6 (25%)
	Hispanic/Latino		25 (59%)	10 (56%)	15 (63%)
	African/ African American		0 (0%)	0 (0%)	0 (0%)
	Asian/Native American/Pacific Islander		3 (7%)	2 (11%)	1 (4%)
	Other		2 (5%)	0 (0%)	2 (8%)
	Consanguinity		1 (2%)	1 (6%)	0 (0%)
Source of nominations	Level IV neonatal intensive care unit		23 (55%)	10 (56%)	13 (54%)
	Cardiovascular Intensive care unit		4 (10%)	2 (11%)	2 (8%)
	Regional paediatric intensive care unit		3 (7%)	1 (6%)	2 (8%)
	Inpatient gastroenterology		9 (21%)	4 (22%)	5 (21%)
	Inpatient neurology		2 (5%)	1 (6%)	1 (4%)
	Inpatient haematology		1 (2%)	0 (0%)	1 (4%)
Birth characteristics	Gestational age	< 37 wks	11 (26%)	5 (28%)	6 (25%)
	Birth weight	< 2.5 Kg	10 (24%)	3 (17%)	7 (29%)
		Not recorded	4 (10%)	1 (6%)	3 (13%)
	APGAR score	at 1 min (average)	6.8	5.4	5
		at 5 min (average)	8.2	7.0	6.5
		<7 at 1 min	14 (33%)	6 (33%)	8 (33%)
		<7 at 5 min	5 (12%)	1 (6%)	4 (17%)
		Not recorded	14 (33%)	4 (22%)	10 (42%)
	Symptom onset	<1 month	36 (86%)	17 (94%)	19 (79%)
Primary system involved	Multiple congenital anomalies		12 (29%)	6 (33%)	6 (25%)
	Neurological		9 (21%)	5 (28%)	4 (17%)
	Hepatic		8 (19%)	3 (17%)	5 (21%)
	Cardiac		3 (7%)	0 (0%)	3 (13%)
	Haematological		3 (7%)	0 (0%)	3 (13%)
	Gastrointestinal		3 (7%)	2 (11%)	1 (4%)
	Endocrine/biochemical		2 (5%)	1 (6%)	1 (4%)
	Musculoskeletal		1 (2%)	1 (6%)	0 (0%)
	Pulmonary		1 (2%)	0 (0%)	1 (4%)
Medical management	Inotropic support		17 (40%	7 (39%)	10 (42%)
	Respiratory support		32 (76%)	12 (67%)	20 (83%)
	Intubated		25 (60%)	8 (44%)	17 (71%)
	Antimicrobial treatment		34 (81%)	14 (78%)	20 (83%)
	>5 Subspecialist consults		13 (31%)	7 (39%)	6 (25%)
Mortality			9 (21%)	2 (11%)	7 (29%)

^a^Three patients received partial diagnoses

While disease phenotypes were typically present at birth, the median age at nomination was 62 days (range 1–301 days; Table S5). The median time from admission to nomination was 5 days. The range of the interval between admission and nomination varied from nomination at a prior admission (in two infants) to 194 days. The median enrolment interval was 5 days (range 0–62 days), and enrolment–receipt of proband blood sample was zero days (range 0–6 days).

### Diagnostic sensitivity of rWGS and standard of care

rWGS diagnosed 19 genetic diseases in 18 of 42 infants (Fig. 1, table S3). Of the patients who received a genetic diagnosis only 3 (6002, 6018 and 6024) had their phenotypes only partially explained by the genetic findings. The remaining 16 diagnoses completely explained the phenotype. All were confirmed with standard genetic tests. Concomitantly, 33 (79%) of 42 infants received 144 standard genetic tests, as clinically indicated (table S6). The most common was chromosomal microarray (CMA) (18 infants, 43%). The diagnostic sensitivity of standard genetic tests (10%) was significantly less than rWGS (43%) (*P* = .0026). rWGS identified all diagnoses made by standard genetic testing. In addition, one infant was incidentally diagnosed with adenovirus infection by metagenomic analysis of WGS (Supplemental Methods). Thus 19 (45%) infants received etiologic diagnoses by rWGS. No diagnoses were considered false positives by clinicians (diagnostic specificity 100%).

The most common presentations receiving diagnoses were multiple congenital anomalies (six (50%) of 12 were diagnosed), and neurologic (five (56%) of nine were diagnosed; Table 1). No demographic or clinical characteristic was significantly associated with diagnosis.

In seven (17%) very ill infants, rWGS was performed with two day time to interpretation. The remaining 35 (83%) families received rWGS using less costly methods with five days to interpretation. In six families (33% of positive results) WGS established a diagnosis for which a specific treatment was available to prevent morbidity or mortality, thus prompting results to be verbally, provisionally reported ahead of medically accepted confirmatory testing. All were subsequently confirmed, with specific therapy initiated before this confirmation.

### Clinical utility

Specific changes in medical or surgical treatment occurred as a result of molecular diagnoses (clinical utility) in 13 (31%) of 42 infants receiving rWGS (including all who received provisional diagnoses, Table 2). Conversely only one child (2%) received changes in care from standard genetic diagnostics (*P* = 0.0015). Seven medications were started in five infants, and medications were discontinued in two (excluding discontinuation of empiric antibiotics prescribed in 82% of infants, Table 1). For example, infant 6056, was diagnosed with megacystic microcolon intestinal hypoperistalsis syndrome, which is lethal in most infants.^10^ Treatment with cisapride resulted in marked improvement in gut motility, such that intestine transplant may not be necessary.^11^ In four infants (22% of diagnosed), surgical procedures were changed. Three were major surgeries. In toto, rWGS-predicated precision medicine was judged to have prevented morbidity in eleven (61%) of eighteen diagnosed infants, compared with none by standard of care (Table 2). Four infants (22% of diagnosed) avoided major morbidity (discussed below; Table 2). Mortality through median day of life (DOL) 430 was two (11%) of 18 diagnosed by rWGS (infant 6012 and 6034), compared with seven (29%) of 24 infants who did not receive diagnoses (Table 1, S4). Mortality was bimodal, with four deaths at DOL 29–35, and five at DOL 209–561.

**Table 2:** Acute precision medicine interventions in thirteen of eighteen infants receiving genetic disease diagnoses and the resultant changes in outcomes

Infant ID	Causal gene	Medication change	Change in surgery	Palliative care initiated	Imaging or procedure change	Morbidity avoided	Mortality avoided
6011	*NPC1*	Miglustat started				Neurologic damage delayed	–
6012	*ARID1B*			Yes		Further futile intensive care	–
6014	*NEB*		Avoided muscle biopsy		Avoided EMG and NCS	Anaesthesia and muscle biopsy	–
6018	*POLR1C*				MRI of brain recommended		–
6019	*GABRA1*	Steroids weaned; confidence in therapy when readmitted			Avoided repeat EEG	Discontinuation of appropriate anti-epileptic at next admission	–
6020	*TPM1*		Cleared for cardiac transplant			Delay in heart transplant	–
6021	*PCDH19*	Ganaxolone started; confidence in medications for child and sibling					–
6024	*PHEX*	Start phosphate and high-dose calcitriol				Development of rickets	–
6026	*JAG1*		Avoided Kasai hepatoportoenterostomy			Kasai and liver transplant	83–94% decrease
6030	*NF1*				Brain MRI for tumour evaluation and MR angiography of renal arteries for stenosis	Potential early detection of *NF1* associated tumors	–
6041	*KCNQ2*	Carbamazepine started; phenobarbital weaned				Prolonged uncontrolled seizures with potential neurological damage	–
6053	*ABCC8*		Earlier partial pancreatectomy			3 additional weeks of hypoglycaemia with potential neurological damage	–
6056	*ACTG2*	Started cisapride					
Total		5 (28%)	4 (22%)	1 (6%)	4 (22%)	10 (56%)	1 (6%)

### Impact of rWGS-associated precision medicine on healthcare utilization

It has been previously shown that WGS, while an expensive test, may actually decrease overall cost (particularly downstream cost) of diagnostic evaluations of medically complex children.^12^ Although also appearing to reduce rather than increase costs in our cohort, the impact of precision medicine could not be quantified in seven of 13 infants without a group of matched historical controls and/or long-term follow up (see Supplementary Results). However, the short-term impact on healthcare utilization was quantified in the remaining six infants (Table 3, Fig. 1). Infant 6011 (previously published^13^) was admitted at seven weeks of age with persistent jaundice and poor weight gain. He was nominated for rWGS, but Spanish consent forms had not yet been approved. Despite extensive evaluation, the aetiology of cholestasis was not identified. He was discharged after eight days, but readmitted four days later due to α-fetoprotein levels of >200,000 ng/mL. rWGS revealed a diagnosis of Niemann-Pick disease, type C1 (*NPC1*). He started treatment with miglustat with plans for intrathecal cyclodextran and remains without neurologic symptoms.^13^ Using a modified Delphi method, the consensus of an international panel of paediatricians was that, had rWGS been performed on his first admission, the second admission would have been unnecessary.

**Table 3:** Effect of rWGS-based precision medicine on acute healthcare utilization in six infants and three matched controls

Subject ID	Presentation and modeled change in care	Gene	Time-to-diagnosis, days (method)	Hospital stay, Days	Decreased hospital stay, days (%)	Total cost	Cost avoided
6011	Cholestasis. 1st admision for etiologic Dx	*NPC1*	7 (G)	8	15 (35%)	$ 25,278	$ 27,004
	Cholestasis. 2nd admission for etiologic Dx			15		$ 27,004	
6012	Palliative care started DOL 250	*ARID1B*	26 (G)	250	42 (17%)	$ 1,949,438	$ 327,506
	Palliative care started DOL 292			292		$ 2,276,944	
6014	Hypotonia. Avoided EMG, GA, muscle biopsy	*NEB1*	7 (G)	45	2 (6%)	$ 156,914	$ 9900
Control 1	Electromyogram, GA, muscle biopsy					$ 9900	
6026	Cholestasis and congenital heart disease. Avoided hepatoportoenterosomy	*JAG1*	3 (G)	11	3 (18%)	$ 50,327	$ 131,795
Control 2	Kasai hepatoportoenterostomy					$ 44,451	
Avg cost	Cost of liver transplant × 43% occurrence					$ 87,344	
6041	Seizures. Diagnosis DOL 4	*KCNQ2*	4 (G)	18	41 (69%)	$ 79,675	$ 181,481
	Seizures. Diagnosis DOL 42		42 (S)	59		$ 261,156	
6053	Hypoglycemia. Diagnosis DOL 12	*ABCC8*	7 (G)	10	21 (68%)	$ 59,769	$ 125,514
	Hypoglycemia. Diagnosis DOL 32		28 (S)	31		$ 185,283	
Healthcare savings				398			$ 803,199
Cost of rWGS in 42 families							$ 674,645
Net healthcare savings							$ 128,554

*GA* general anesthesia, *G *rWGS, *GI* inpatient gastroenterology, *S* standard genetic diagnostic test, *Avg* average

Change in Healthcare costs due to whole-genome sequencing. Patient 6011 would have avoided second hospitalization if *NPC1* diagnosis was made on first admission, saving $27,004. Patient 6012 had compassionate withdrawal of care after diagnosis of *ARID1B* negating need for 42 day planned antibiotic course and continued intensive care stay with a projected cost of $327,506. Patient 6014 avoided a muscle biopsy and EMG and likely need for recovery in NICU from anaesthesia due to hypotonia, saving $9900. Patient 6026 avoided the need for a Kasai saving $44,451 and also avoided 43% increased likelihood of needing liver transplant if the Kasai had been performed resulting in net savings of $87,344 ($203,125 (cost of surgery and 90 days post-transplant) × 43%). Patient 6041 had diagnosis made 38 days earlier than case control who had standard work up one year earlier resulting in savings of $181,481. Patient 6053 had diagnosis of *ABCC8* made 21 days earlier than what is the standard from the literature resulting in a net savings of $125,514 from shortened stay in NICU while trying to control severe hypoglycaemia. Total savings was $803,200 which was $128,555 less than actual cost of sequencing all 42 families

Infant 6012 was admitted to the NICU at birth with congenital heart disease (Shone’s complex) and congenital diaphragmatic hernia. She had an extremely complicated course with multiple surgical interventions, including post-cardiac surgery extracorporeal membrane oxygenation, gastrostomy tube placement with Nissen fundoplication, tracheostomy for respiratory failure, and treatment for recurrent sepsis. She was enrolled on DOL 224, shortly after the study commenced. A diagnosis of Coffin-Siris syndrome was reported on DOL 250, at which time she had septic shock, requiring inotropic support with multiple agents. A six week course of intravenous antibiotics had been planned. Upon diagnosis, in light of the prognosis, the family elected for comfort care, and the patient expired that day. Time to death after withdrawal of life-sustaining treatment is not commonly a protracted event.^14,15^ The Delphi panel could not achieve consensus with respect to earlier election of palliative care if the diagnosis had been known soon after birth. However, there was consensus that without rWGS, NICU care and therapy for sepsis would have continued until resuscitation was no longer possible.

Neonate 6014 was admitted to the NICU at birth with respiratory distress. He was hypotonic and aspirated feeds, resulting in gastrostomy tube placement. He was enrolled on DOL 35, and a provisional diagnosis of Nemaline Myopathy was communicated on DOL 42. The panel consensus was that having the molecular diagnosis avoided the need for muscle biopsy, and that it was reasonable to compare projected costs of this procedure (from a matched control infant).

Infant 6026 was admitted to inpatient gastroenterology on DOL 76 with severe cholestasis. He was noted to have congenital heart disease. On DOL 78 he developed respiratory distress and metabolic acidosis, and was transferred to the PICU. He was scheduled for intraoperative cholangiogram with reflex to Kasai hepatoportoenterostomy on DOL 83 for evaluation of suspected biliary atresia (see supplementary results). He was enrolled on DOL 80 and received a provisional diagnosis of Alagille syndrome on DOL 83. This provisional diagnosis was reported to the operating room immediately before induction of general anaesthesia for intraoperative cholangiogram and Kasai procedure. The diagnosis was confirmed by CMA several days later. Control infant 2 was admitted to inpatient gastroenterology with cholestasis one month later, and underwent hepatoportoenterostomy for biliary atresia, costs were included for an expected additional two days of PICU recovery. Estimated rWGS-associated savings were $44,451. Given that 90% of infants with Alagille syndrome have abnormal intraoperative cholangiograms, the panel consensus was that he had 83–94% reduction in mortality as a result of cancellation of hepatoportoenterostomy, and 70–80% reduction in likelihood of liver transplant.^16–18^ Estimates of the cost of a subsequent liver transplant were based on an average charges of $812,500^19^ given the typical 25% cost to charge ratio, estimated costs would be $203,125. Based on two previous publications^16,17^ which showed total of 14 out of 24 patients with Alagille who had a Kasai requiring liver transplant compared with seven out of 46 patients with Alagille syndrome without Kasai requiring liver transplant it was estimated that Kasai increased rate of liver transplant by 43%. Consequently the likelihood weighted saving was $87,344 for liver transplant. Total estimated savings were $131,795.

Neonate 6041 was born by normal delivery at term, and immediately admitted to the NICU for tonic-myoclonic seizures. While seizures were largely controlled by phenobarbital, levetiracetam, and topiramate, she was too sedated to tolerate oral feeds. Upon dose reduction, the seizures returned. Electroencephalogram revealed multifocal, epileptiform abnormalities, and burst suppression. She was enrolled on DOL 1, and a provisional diagnosis of Early Infantile Epileptic Encephalopathy type seven was communicated on DOL 4. Carbamazepine was added to levetiracetam,^20,21^ providing complete seizure control without sedation, and she was discharged home on DOL 19, in time for a family Christmas. Control neonate 3 was admitted to the same NICU sixteen months earlier, with the same presentation and molecular diagnosis. However, he was diagnosed by standard tests, which took an additional six weeks to return, during which he continued to have uncontrolled seizures. He has severe developmental delay. His 59 day hospitalization cost $211,484. An estimate assuming that the actual cost of $4,426/day for Case 6041 would have persisted for the additional 42 inpatient days, produced estimated rWGS-associated cost savings of $181,481. At thirteen months of age, she remains seizure free and has minimal developmental delay. The panel consensus was that she avoided likely neurologic damage associated with prolonged uncontrolled seizures.^20–24^ Cost savings of preventing neurological devastation were not calculated.

Neonate 6053 was transferred to the RCHSD NICU at birth with hypoglycaemia and suggestion that she was the infant of a diabetic mother. She was enrolled on DOL 5, and a provisional diagnosis of focal hyperinsulinemic hypoglycaemia was communicated on DOL 12, indicating the need for surgical evaluation. The median age at surgery for infants with focal hyperinsulinemic hypoglycaemia is 78 days.^25^ Blood sugars remained brittle during the hospitalization. Persistent or recurrent hypoglycaemia in neonates with hyperinsulinemic hypoglycaemia is associated with neurologic damage, epilepsy, and intellectual disability.^9,25–27^ She had surgery on DOL 28, at least 21 days earlier than possible by standard testing.^25^ The panel consensus was that it was reasonable to compare Case 6053 actual utilization (Fig. 1b) vs actual costs plus 21 additional days of NICU stay.

In total, in the six infants, rWGS improved the care of the patients and reduced the costs by at least $803,200 (Table 3).The current full cost of rWGS as a CLIA Laboratory Developed Test, including indirect costs and treatment guidance, was $8482–$22,128 (singleton, duo, trio, and quad, table S4). The total cost of rWGS in 42 families was $674,645. Net of rWGS cost in the whole cohort, inpatient cost was estimated to be reduced by $128,555.

## Discussion

The systematic evidence for clinical utility of rWGS in infants with likely genetic diseases, while dramatic, has been limited.^9,13,28^ Here, we report the impact of rWGS on clinical outcomes and healthcare utilization in 42 infant inpatients. In contrast to two prior studies, enrolees were predominantly Hispanic/Latino, and enrolled from various inpatient settings, with 71% from intensive care units. Forty percent received inotropic cardiac support and 76% received ventilatory support. Severity of illness and mortality were similar to the prior study.^9^ This cohort was well suited to examine clinical utility.

rWGS had a significantly higher diagnostic sensitivity (43%) than standard of care (10%), in agreement with published rates of diagnosis of genetic diseases by WGS (average 40%, range 32–57%).^9,29,30^ Of note, the diagnostic sensitivity was high despite broader enrolment than prior studies.^9^ This supports the diagnostic sensitivity of rWGS as a first tier test in a substantial subset of infants admitted to children’s hospitals for reasons other than prematurity.^29,31^

The types of genetic diagnoses herein were similar to prior studies of symptomatic infants:^7,9,32^ Autosomal dominant disorders were the most common, and causative variants were de novo as frequently as inherited. There were no recurrent diagnoses, reflecting genetic heterogeneity in inpatient infants.

From blood sample receipt to provisional diagnosis, rWGS is possible in 26–48 h. Herein, however, median time from enrolment to final report in the medical record was 23 days (range 5–69 days), similar to prior studies.^9^ Two factors slowed diagnoses: first, confirmatory clinical testing was required before final reporting (adding up to 16 days to time-to-report). Secondly, not all rWGS was performed with the fastest methods, since it was more than two-fold more expensive. In six acutely ill infants (33% of diagnoses), provisional diagnoses were communicated before confirmatory testing (median 7 days, range 3–12 days), since treatments existed that could reduce morbidity or mortality. In the prior two studies, only one infant met these criteria (3% of diagnoses), and diagnosis was in 3 days.^9^ The clinical utility of rWGS was measured by acute precision medicine interventions, defined as a change in management which physicians agreed resulted directly from the rWGS diagnosis and that occurred promptly after reporting. Exclusive of changes in genetic counselling, subspecialty consultation, or empiric antibiotics,13 (72%) of 18 rWGS diagnoses (31% of 42 infants tested) received precision medicine. This concurred with prior studies.^9,31^ In a paired test, the rate of clinical utility was significantly greater by rWGS (33%, 11 of 33) than by standard of care (2%, *P* = 0.0015).

The most important measure of clinical utility is improved outcomes. Herein, rWGS-based precision medicine was adjudged to avoid morbidity in 26% (11) of 42 infants, major morbidity in four (10%), and reduced likelihood of acute mortality by 43% in one (2%). Two prior studies reported acute health outcomes of rWGS or exome-based precision medicine in infants. They found that major morbidity was avoided in 3% (four) of 115 infants, and mortality in 3% (three).^9,31^ Two diseases identified in these studies were also observed in this series, with the same avoided morbidity: one was recurrent hypoglycaemia due to focal type 1 familial hyperinsulinism. rWGS enabled expedited partial pancreatectomy, avoiding hypoglycaemic neurologic damage.^9,25–27^ The other was Ohtahara syndrome, where rWGS enabled complete control of seizures without sedation, potentially avoiding neurologic damage.^9,20–22,24^ Thus, the magnitude and types of impact of rWGS on outcomes in inpatient infants was replicated in Hispanic/Latino infants in urban southern California.

In addition, the prior study showed rWGS to aid palliative care decisions.^9^ Herein, diagnosis led to palliative care in one (6%) of 18 infants, less than that study (six (30%) of 20 diagnoses; *P* = 0.09).

When considering introducing new methods in healthcare, improved clinical outcomes are the most important factors. However, these outcomes must be considered in the context of health care utilization and cost. To date, studies of genome-wide sequencing have limited financial modelling to compare with the cost of standard genetic tests.^32^ Here, we examined healthcare utilization associated with precision medicine interventions in six infants (46% of 13 receiving rWGS-associated precision medicine). rWGS was estimated to reduce the length of stay by 124 days, and inpatient professional and facility cost by at least $803,200. These estimates are very conservative and reflect the judgement of a Delphi panel (Supplemental Methods). For the palliative care patient, the parents elected to end heroic measures once they were aware of the genetic diagnosis. Had this finding occurred earlier in the patient’s care it is possible palliative care would have started earlier saving an additional $1.2 million. This would have made gross savings from the study $2 million. In addition the diagnoses that were made could have very likely been made with proband only analysis which would have yielded a cost of $356,244 for the sequencing.

There were several limitations to the cost analysis. First, it was based on only six “case” infants, two historical “controls”, and published literature values. However, since preparation of this manuscript, we have studied the clinical utility of rWGS in an additional ~250 infants. Amongst these, we have had two additional diagnoses of early infantile epileptic encephalopathies in neonates with status epilepticus, in which diagnosis resulted in targeted therapy and prompt cessation of seizures akin to case 6041. We have had a second diagnosis of nemaline myopathy in a neonate with hypotonia, who avoided a muscle biopsy akin to case 6014. Second, consensus of a physician panel and the Delphi method were used to judge the impact of precision medicine, and may have been incorrect. Third, utilization was only calculated during that hospitalization, with the exception of a rapid re-admission in infant 6011, and a liver transplant in infant 6026. Children with genetic disorders have substantial ongoing healthcare utilization. We did not include the likely avoidance of a gut transplant in infant 6056, nor avoidance of neurologic damage in infant 6041. Further, infant genetic diseases exert profound emotional, financial, social, and physical stress within families. These include parental divorce, depression and anxiety, and sibling behavioral, developmental, and persistent health complications. The costs of these complications were omitted. Finally, palliative care is not uncommon in regional NICUs, and decisions are often delayed by absence of etiologic diagnoses.^7^ We did not estimate the optimal rWGS-associated precision medicine scenario, in which rWGS would have been ordered at time of admission.

In conclusion, rWGS is a unique, high-cost healthcare innovation that appears to improve healthcare outcomes while decreasing cost of care. While rWGS merits consideration as a first tier test in a subset of acutely ill inpatient infants, further studies are needed to delineate clinical presentations for which outcomes are consistently improved and optimal timing of rWGS orders and time-to-result.

## Methods

Full methods are described in the Supplementary Material.

### Study design

Retrospective comparison of clinical utility, outcomes, and healthcare utilization of rWGS and standard of care (including genetic testing) was approved by the institutional review board (IRB) at Rady Children’s Hospital-San Diego (RCHSD) and the Food and Drug Administration (FDA; ClinicalTrials.gov NCT02917460; figure S1). Inpatient infants at RCHSD without etiologic diagnoses, in whom a genetic disorder was possible, were nominated by diverse clinicians from 26 July 2016–8 March 2017. Informed consent was obtained from at least one parent or guardian.

### rWGS, interpretation, and reporting

Clinical features were extracted from electronic medical records (EMR, table S1) and mapped to genetic diagnoses.^13,28^ Trio blood samples were obtained where possible. rWGS was performed to ~45-fold coverage as previously described.^13,28^ Structural variants were identified with Manta and CNVnator, a combination that provided the highest sensitivity and precision on 21 samples with known structural variants.^33,34^ Structural variants were filtered to retain those affecting coding regions of known disease genes and with allele frequencies <2% in the RCIGM database. If WGS established a diagnosis for which a specific treatment was available to prevent morbidity or mortality, this was immediately conveyed to the clinical team. All causative variants were confirmed by Sanger sequencing or CMA, as appropriate. Secondary findings were not reported.

### Clinical utility and healthcare utilization

Acute clinical utility of diagnoses and impact on outcomes were evaluated by EMR review, interviews with clinicians, published values, and consensus of at least two paediatricians, one of whom was a relevant paediatric subspecialist and one a medical geneticist. Outcomes were assessed until 9 October 2017.

The effect on healthcare utilization was modelled in six infants in whom diagnoses changed treatment and outcomes, by comparing actual healthcare utilization with that of a counterfactual diagnostic trajectory. A modified Delphi method was used to establish consensus for counterfactual trajectories.^35^ In five infants, the counterfactual trajectory was molecular diagnosis following standard testing based on recent control subjects from the same NICU prior to rWGS and/or literature values. Impact was calculated by the proportionate increase pain length of stay multiplied by the actual average daily utilization after one off tests such as MRI scans were excluded. In one infant we considered a counterfactual trajectory as if rWGS was ordered during a prior admission, with correspondingly earlier implementation of precision medicine. In this infant, retrospective impact was calculated by avoidance of the second admission. The cost of rWGS was calculated, including pretest consultation, Sanger confirmation, counselling, result disclosure, and precision medicine guidance (table S4).

### Statistical analysis

Diagnostic sensitivity and the rate of clinical utility for standard care and rWGS were compared using McNemar’s test for paired nominal data. Fisher’s exact test was used to compare the rate of palliative care initiation with another study. Two-tailed *p*-values less than 0.05 were considered statistically significant.

### Data and material availability

Data are available at LPDR (https://www.nbstrn.org/research-tools/longitudinal-pediatric-data-resource).

## Electronic supplementary material

Supplemental Material File
