# Supplementary material for: Rapid whole-genome sequencing decreases infant morbidity and cost of hospitalization
Source: NPJ Genom Med. 2018 Apr 4;3:10. doi: 10.1038/s41525-018-0049-4 (PMC5884823; doi:10.1038/s41525-018-0049-4)
Supplement: Supplementary file 1 — Supplemental Material File [file 41525_2018_49_MOESM1_ESM.docx]

**Supplementary Material**

**Precision Medicine through Rapid Whole Genome Sequencing Decreases Morbidity and Healthcare Utilization in Inpatient Infants**

Lauge Farnaes MD PhD^1,2,†^, Amber Hildreth DO^1,2,†^, Nathaly M. Sweeney MD MPH^1,2,†^, Michelle M. Clark PhD^1^, Shimul Chowdhury PhD^1^, Shareef Nahas PhD^1^, Julie A. Cakici BSN^1^, Wendy Benson MBA^1^, Robert H. Kaplan PhD^3^, Richard Kronick PhD^4^, Matthew N. Bainbridge PhD^1^, Jennifer Friedman MD^1,2,6^, Jeffrey J. Gold MD PhD^1,6^, Yan Ding MD^1^, Narayanan Veeraraghavan PhD^1^, David Dimmock MD^1^, and Stephen F. Kingsmore MB ChB BAO DSc^1^ on behalf of the RCIGM Investigators.

Affiliations:

1. Rady Children’s Institute for Genomic Medicine, San Diego, CA, USA;
2. Department of Pediatrics, University of California San Diego, San Diego, CA, USA;
3. Torrey Pines Health Group Inc., San Diego, CA, USA;
4. Department of Family Medicine and Public Health, University of California San Diego, San Diego, CA, USA;
5. Department of Neurosciences, University of California San Diego, San Diego, CA, USA;

† Contributed equally to this work.

**Correspondence:** S Kingsmore ([skingsmore@rchsd.org](mailto:skingsmore@rchsd.org)), (858) 966-4998. Rady Children’s Institute for Genomic Medicine, Rady Children’s Hospital, 3020 Children’s Way, San Diego, CA 92113, USA

The RCIGM investigators are Jaime Barea, Sergey Batalov, Jeanne Carroll, Sarah Caylor, George Chiang, Casey Cohenmeyer, Nicole G. Coufal, Marva Evans, Joseph Gleeson, Jose Honold, Farhad B. Imam, Amy Kimball, Brian Lane, Crystal Le, Sandra Leibel, Laurel Moyer, Paulina Ordonez, Julie Ryu, Mark Speziale, Denise Suttner, Charles Sauer, Richard Song. Lisa Salz, Kristen Wigby, Audra Wise, Meredith Wright,

**Funding**: Grant U19HD077693 from NICHD and NHGRI.

† Contributed equally to this work.

**Supplemental Methods………………………………………………………………………………………………………………………………..Page 2**

**Supplemental Results…………………………………………………………………………………………………………………………………..Page 9**

**Supplemental References.……………………………………………………………………………………………………………………………Page 11**

**Table S1……………………………………………………………………………………………………………………………………………………….Page 11**

**Table S2……………………………………………………………………………………………………………………………………………………….Page 17**

**Table S3……………………………………………………………………………………………………………………………………………………….Page 18**

**Table S4……………………………………………………………………………………………………………………………………………………….Page 19**

**Figure S1…………………….………………………………………………………………………………………………………………………………..Page 20**

**Figure S2………………………………………………………………………………………………………………………………………………………Page 21**

**Supplementary Methods**

**Study Design**

Retrospective comparison of clinical utility, outcomes, and healthcare utilization of rapid whole genome sequencing (rWGS) and standard of care (including clinical genetic testing) was approved by the institutional review board (IRB) at Rady Children’s Hospital-San Diego (RCHSD)/University of California-San Diego (ClinicalTrials.gov NCT02917460) and the Food and Drug Administration (FDA). Inpatient infants at RCHSD without etiologic diagnoses, and in whom a genetic disorder was possible, were nominated by diverse clinicians from July 26 2016–March 8 2017 (figure S1). Informed consent was obtained from at least one biological parent, or guardian.

**rWGS, Interpretation and Reporting**

Clinical features of infants were extracted from electronic medical records (EMR), translated into human phenotype ontology terms (table S1), mapped to all genetic diagnoses, and rank ordered by goodness of fit with Phenomizer or Phenolyzer^1,2^. Blood samples were obtained from probands, and parents (trios), where possible. Blood samples from inpatient infants were obtained within the maximum allowable daily phlebotomy, and minimum haemoglobin in infants with respiratory or cardiovascular compromise. DNA was isolated using standard methods and WGS libraries were prepared with PCR-free methods (Illumina, San Diego, CA) as described^3^. rWGS was performed at Envision Inc. (Huntsville, AL) for the first 27 families by 45-fold 2 x 150 nucleotide (nt) sequencing on Illumina HiSeq X instruments (5-10 day turnaround). Remaining families were sequenced in house in two modes^4^: In very ill infants, 2 x 100 nt proband rWGS was performed on HiSeq 2500s in rapid run mode. Other rWGS was 2 x 150 nt on a HiSeq 4000. Rapid alignment and variant calling was by Dragen (Edico Genome, San Diego, CA; table S2)^3^. Variants were annotated, analysed, and interpreted with Opal Clinical (Fabric Genomics, Oakland, CA)^5,6^. Causative variants were confirmed by Sanger sequencing. Secondary findings were not reported.

**Metagenomic analysis of WGS**

Unmapped reads were identified in the WGS BAM file and then realigned against a collection of 3932 viral genomes obtained from NCBI.  Alignments were conducted using BWA.  To prevent numerous, non-specific matches to low complexity regions, reads were filtered for perfect matches (i.e. all 100 or 150bp of the read mapped perfectly to the viral genome).  Using parental genomes as controls, we established the number of reads we would expect to map to any particular genome by chance.  Samples were considered positive for a virus if the number of reads aligning to the viral genome was 4 standard deviations above the control average.

**Clinical Utility, Healthcare Utilization**

The acute clinical utility of rWGS-based diagnoses (i.e. short-term implementation of precision medicine interventions) and impact on outcomes were evaluated by EMR review, interviews with clinicians, published values, and evaluation by at least two paediatricians, of whom one was a relevant paediatric subspecialist and one a medical geneticist.

The effect on healthcare utilization was modelled in six infants in whom rWGS diagnoses changed management and outcomes quantitatively. In these infants, we calculated actual healthcare utilization and that of either/both of two counterfactual diagnostic scenarios. First, we measured actual healthcare utilization with rWGS, with the actual time-to-diagnosis, implementation of precision medicine, hospital course, and outcome. The length of hospital stay, actual physician worked relative value units (wRVUs), and cost of inpatient care were measured. The cost of trio rWGS was calculated, including consultation for pretest assessment, counselling, result disclosure, and precision medicine guidance (Table S4). We included the cost of trio Sanger sequencing to validate variants detected by rWGS. Second, in four infants, we considered a counterfactual standard diagnostic pathway without rWGS, in which a molecular diagnosis was made with standard tests and standard time-to-diagnosis, in which implementation of precision medicine was correspondingly delayed (Table S5). In three of the four, prospective estimates of resource utilization were based on matched, historical control subjects from the same NICU with standard time-to-diagnosis. In two of the four, counterfactual resource utilization was estimated based on literature values for time-to-diagnosis, and modelled prospective impact based on proportionately increased inpatient days and the average daily utilization during that hospitalization. Thirdly, in two infants we considered the counterfactual scenario wherein rWGS was ordered earlier – shortly after NICU admission – with correspondingly earlier implementation of precision medicine. In these infants, retrospective impact was estimated based on proportionately decreased inpatient days and the average daily utilization during that hospitalization.

Facility costs are estimated by multiplying hospital charges by the estimated cost to charge ratio supplied by Rady Children’s Hospital Chief Financial Officer. Professional costs are estimated by multiplying professional charges by the estimated average payment to charge ratio for professional services.

**Delphi Method**

A modified Delphi method was used to establish consensus for the counterfactual trajectories. An international panel of paediatricians representing a variety of subspecialties that covered those typically involved in quaternary neonatal care was assembled. Panellists were required to not be currently funded by RCIGM or RCIGM investigators or have any other financial ties to the institute. The panel consisted of seven paediatric clinical geneticists, two hospital-based paediatricians, two paediatric neurologists, and two neonatologists, from Australia (two), United Kingdom (one) and the United States (ten).

The panel developed consensus on expected care using the Delphi Method, a consensus method developed to utilize expert opinion to make a knowledge-based decision when insufficient information is available. This method is increasingly used to develop consensus-based guidelines in medicine and rare diseases. Expert panellists review and summarize the available knowledge and answer surveys concerning the issues in question. The survey is scored to determine the variation in opinion; if consensus is not reached, these items are returned to the panellists for a second round, this time with the mean of responses from the first round available.

The survey instrument comprised a five-point bipolar Likert scale. The range of potential responses included strongly disagree, disagree, neutral, agree or strongly agree. Experts were allowed to note that they did not have sufficient expertise to answer a question, and their score was then excluded. Each answered response was assigned a numerical score, such that strongly disagree was scored as 1, disagree was scored as 2, neutral as 3, agree as 4 and strongly agree as 5. The mean consensus score for each item was then tallied. Items with a mean consensus score of > 4 (Agree/Strongly Agree) OR <2 (Disagree/Strongly Disagree were considered to reach consensus (as Agree or Disagree, respectively).

For items that did not meet consensus, a survey of these questions was returned to the panellists for a second round, with items marked with the group’s score so that each panellist was aware of the group mean when they re-scored the survey. Following completion of all surveys, a decision was reached to discard those recommendations that did not meet consensus. Only items that met consensus (average scores of > 4) were included in the formal conclusions.

**Case Reports for Delphi Method**

**Newborn with Ohtahara syndrome**

This was born at 37w 3/7d via spontaneous vaginal delivery. At ~16 hours of life, the newborn was noted to have bilateral tonic and clonic movements, and the patient was transferred to a regional NICU. Video Electroencephalogram, placed due to the suspicion for seizures, showed discontinuous background with burst-suppression type pattern and near continuous epileptiform discharges, consistent with Ohtahara syndrome. Temporary seizure control was obtained by phenobarbital, levetiracetam and topiramate regimen. The infant, however, was overly sedated and could not tolerate oral feeds. The patient was, therefore, weaned off phenobarbital and topiramate, but the seizures returned. A de novo variant of *KCNQ2* gene (c875T>C, p.L292P (Ref Seq: NM_172107.3:c.875T>C) was identified within six days of life by the RCIGM’s rapid whole genome sequencing (rWGS) program. Given the *KCNQ2* variant, carbamazepine was added to levetiracetam. This dual AED combination lead to complete seizure control without over sedation and patient was discharged at day of life 19. At eight months of age, she remains seizure free and has minimal/no developmental delay.

For comparison a similar patient was admitted to the same NICU at birth for seizures with a different *KCNQ2* variant 1 year prior to offering rWGS, and was treated by the same neonatologist and neurologist. Seizures were also stopped with carbamazepine when the standard molecular testing results were returned, 6 days after being sent. The child was discharged on DOL59 and has severe developmental delay.

*KCNQ2* is associated with 2 distinct phenotypes: a benign neonatal seizure phenotype and an Ohtahara syndrome phenotype. “Early recognition of *KCNQ2* encephalopathy followed by the most appropriate and effective treatment may be important for reducing the neurodevelopmental impairment associated with this disorder. Twelve patients had moderate-to-severe developmental delay at follow-up. However, the two patients whose seizures ceased within a few days of onset showed only mild cognitive impairment.”^7^

**Delphi round 2 are the following reasonable questions?**

Given the strong overlap in clinical presentation and same disease gene it is reasonable to use these 2 cases for comparison?

Strongly disagree, Disagree, Neutral, Agree, Strongly agree, Unable to comment. Round 2 consensus: Agree.

It is reasonable to attribute the improved long term outcomes to the earlier diagnosis and earlier complete control of seizures?

Strongly disagree, Disagree, Neutral, Agree, Strongly agree, Unable to comment. Round 2 consensus: Agree.

**11 week old male admitted for work up of neonatal cholestasis**

Patient was born at term without complications. He was admitted to inpatient gastroenterology on DOL 76 with severe cholestasis and failure to thrive. He was noted to have a flow murmur that was confirmed by echocardiogram to be peripheral pulmonic stenosis. On DOL 78 he developed respiratory distress and metabolic acidosis, and was transferred to the PICU. Liver biopsy demonstrated giant cell hepatitis (although it was a small sample and difficult to interpret further information). HIDA scan was non-excreting. He was enrolled on DOL 80 for rWGS. He was scheduled for a laparotomy with Intra-operative cholangiogram with reflex to Kasai hepatoportoenterostomy on DOL 83 for treatment of a clinical diagnosis of biliary atresia. He received a provisional diagnosis of Alagille syndrome on DOL 83 when he was in the operating room immediately before induction of general anaesthesia.

**Delphi round 2 are the following reasonable questions?**

Given that surgery was halted when the child was in the operating room is it reasonable to assume that this prevention of surgery was as a direct result of the molecular diagnosis?

Strongly disagree, Disagree, Neutral, Agree, Strongly agree, Unable to comment. Round 2 consensus: Agree.

Data suggests that 90% of children with Alagille syndrome have abnormal intraoperative cholangiograms^8^. Given the non-excreting HIDA scan, is it reasonable to assume that this child had 90% chance of proceeding to a Kasai procedure?

Strongly disagree, Disagree, Neutral, Agree, Strongly agree, Unable to comment. Round 2 consensus: Agree.

Published data in one case series^9^ report that liver transplantation was performed in 100% of 15 children with Alagille syndrome who had been misdiagnosed and received a Kasai procedure, but only 20% of children with Alagille who did not have a Kasai. Mortality was observed in 60% among the Alagille Kasai group, and 10% among the non-Kasai group. Another series^10^ suggest that 47% of 19 Alagille syndrome infants who received a Kasai procedure and 14% of 36 matched Alagille controls who did not receive a Kasai procedure needed a liver transplant. Mortality was observed in 32% among the Alagille Kasai group, and 2% among the Alagille non-Kasai group. Is it reasonable to assume that cancellation of the Kasai procedure in this child was associated with an 83% (60-50/60) to 94% (32-2/32) reduction in likelihood of mortality and a 70% (47-14/47) to 80% (100-20/100) reduction in risk of having a liver transplant?

Strongly disagree, Disagree, Neutral, Agree, Strongly agree, Unable to comment. Round 2 consensus: Agree.

Is it reasonable to include saved costs from an avoided liver transplant as an outcome from WGS?

Strongly disagree, Disagree, Neutral, Agree, Strongly agree, Unable to comment. Round 2 consensus: Agree.

**5 day old with hypoglycaemia**

The case was born large for gestational age (3.9 Kg) at 37 weeks and 1 day. At 8 hours of life was noted to be cyanotic and feeding poorly. Blood glucose measured at 12mg/dL (0.66mmol/l) initially and patient required multiple D10 boluses to improve blood glucose to 60 mg/dL (3.33mmol/l). A critical sample was drawn showing hyperinsulinemia (522) with glucose at 55 (3.05 mmol/l), cortisol 4.1 mcg/dL (113 nanomoles/L) (not as high as expected). Growth hormone, betahydroxybuterate, free fatty acids, and thyroid hormones were all normal. There was a history of possible maternal diabetes. Mother strongly believed the low blood sugars were all due to maternal diabetes and was not a genetic or other disorder.

She was enrolled on DOL 5, and a provisional diagnosis of focal hyperinsulinemic hypoglycaemia was communicated on DOL 12 (Dominant (paternal uniparental isodisomy) stop gain variant in *ABCC8*). This result indicated referral for surgery. The median age at surgery for infants with focal hyperinsulinemic hypoglycaemia is 78 days^11^. She was referred for surgery at least 21 days earlier than would have been possible by standard testing. When provided with molecular results parents agreed to transfer to centre for surgical evaluation within 6 hours of receiving result.

Outcome: This allowed transfer to another facility for definitive treatment by near total pancreatectomy. The patient had surgery at 28 days of life. The standard time to surgery is 78 days for infants with focal hyperinsulinemic hypoglycaemia^11^. The patient was estimated to have had at least a 3 week reduced stay in the NICU. Blood sugars remained brittle during the hospitalization. Persistent or recurrent hypoglycaemia in neonates with hyperinsulinemic hypoglycaemia is associated with neurologic damage, epilepsy, and intellectual disability^12,13^.

**Delphi round 2 are the following reasonable questions?**

It is reasonable to infer that the molecular test was what precipitated the parental change of assessment?

Strongly disagree, Disagree, Neutral, Agree, Strongly agree, Unable to comment. Round 2 consensus: Agree.

It is reasonable to assume that this change of point of view lead to an earlier transfer?

Strongly disagree, Disagree, Neutral, Agree, Strongly agree, Unable to comment. Round 2 consensus: Agree.

Using published dates of time to surgery is an appropriate timeframe for estimating reduction in time to surgery?

Strongly disagree, Disagree, Neutral, Agree, Strongly agree, Unable to comment. Round 2 consensus: Agree.

It would be more appropriate to model a reduction in hospital stay based on the timeframe for return of conventional molecular results, assuming a decision to go to surgery would be made immediately upon return of conventional test results?

Strongly disagree, Disagree, Neutral, Agree, Strongly agree, Unable to comment. Round 2 consensus: Agree.

We should include the potential avoidance of neurologic damage from prolonged refractory hypoglycaemia?

Strongly disagree, Disagree, Neutral, Agree, Strongly agree, Unable to comment. Round 2 consensus: Neutral (3.92).

**Infant with Shone’s complex, congenital diaphragmatic hernia, and recurrent sepsis**

Infant was transferred to the RCHSD NICU at birth with congenital heart disease (Shone’s complex) and congenital diaphragmatic hernia. She had an extremely complicated course with multiple surgical interventions, including post-cardiac surgery Extracorporeal Membrane Oxygenation, gastrostomy tube placement with Nissen fundoplication, tracheostomy for respiratory failure, recurrent infections and developmental delay. She was enrolled on DOL 224, shortly after the study commenced, and received a final diagnosis of Coffin-Siris syndrome on DOL 250.

At time of diagnosis she was in septic shock, requiring inotropic support with multiple agents. Upon diagnosis, medical geneticists met with the family. In light of the prognosis, the family elected palliative care including allowing a natural death. The child died that same day.

Published data suggests that following a fatal diagnosis parents elect to withdraw care within 5 days of diagnosis^14^ ^15^. The parents in this situation stated that knowing that there child wasn’t going to improve was enough for them to make a decision to withdraw care and did so within 6 hours of receiving the molecular diagnosis.

Coffin-Siris is associated with significantly increased risks of recurrent pulmonary infections which are significant in the face of a diaphragmatic hernia. However it is hard to argue that Coffin-Siris in and of itself is a terminal diagnosis.

**Delphi round 2 are the following reasonable questions?**

A diagnosis of Coffin-Siris in addition to the child’s other significant problems would have been sufficient to have made a decision to recommend to the parents not repair the diaphragmatic hernia?

Strongly disagree, Disagree, Neutral, Agree, Strongly agree, Unable to comment. Round 2 consensus: Neutral (3.25).

A diagnosis of Coffin-Siris in addition to the child’s other significant problems would have been sufficient to have made a decision to recommend to the parents not repair the Shone’s complex?

Strongly disagree, Disagree, Neutral, Agree, Strongly agree, Unable to comment. Round 2 consensus: Neutral (3.42).

A diagnosis of Coffin-Siris in addition to the child’s other significant problems would have been sufficient to have made a decision to recommend to the parents not to put the child on ECMO the first or second time?

Strongly disagree, Disagree, Neutral, Agree, Strongly agree, Unable to comment. Round 2 consensus: Neutral (3.67).

The child had just been diagnosed with septic shock and was receiving several inotropic agents for circulatory support. A six week course of intravenous antibiotics had been planned. It is reasonable to suppose that without the diagnosis the clinical team would have continued to treat this child for sepsis?

Strongly disagree, Disagree, Neutral, Agree, Strongly agree, Unable to comment. Round 2 consensus: Agree.

Given the history of the child’s clinical presentation, it is reasonable to assume that the child would have continued care in the NICU until a diagnosis was obtained or resuscitation was no longer possible?

Strongly disagree, Disagree, Neutral, Agree, Strongly agree, Unable to comment. Round 2 consensus: Agree.

**7 week old male admitted for work up of neonatal cholestasis**

Patient was born full term via caesarean-section. Parents had noticed jaundice since birth. He was admitted, at seven weeks of age, for further work up after outpatient labs revealed elevated hepatic transaminases, elevated direct bilirubin, and failure to thrive. On exam he was hypotonic with hepatosplenomegaly and clinodactyly. Initial liver biopsy suggested giant cell hepatitis. He was nominated for rWGS, but Spanish consent forms had not yet been approved. Despite extensive evaluation, the aetiology of cholestasis was not identified. His labs stabilized so he was discharged home after eight days.

However, he was readmitted four days later due to alpha fetoprotein levels of >200,000 ng/mL (indicating progressive inflammation). This child received rWGS on the second admission in which a liver biopsy was performed to assess liver disease.

Molecular Diagnosis: Homozygous *NPC1* c.2713 C>T (p.Gln905Ter). WGS results obtained 16 days prior to clinical gene panel testing^5^.

**Delphi round 2 are the following reasonable questions?**

It is reasonable to assume the child would not have been readmitted for inpatient management of rising AFP levels if a diagnosis of Niemann-Pick C1 had been made on the first admission?

Strongly disagree, Disagree, Neutral, Agree, Strongly agree, Unable to comment. Round 2 consensus: Agree.

It is reasonable to assume that this second liver biopsy would not have been performed if the child had a molecular diagnosis from the first admission?

Strongly disagree, Disagree, Neutral, Agree, Strongly agree, Unable to comment. Round 2 consensus: Agree.

**5 week old male referred to us from the NICU for hypotonia and aspiration**

The case was born at full term by caesarean-section and was noted to have respiratory distress at birth. He was transferred to the NICU after birth and was noted to be hypotonic. He was noted to have pooling of secretions and aspiration with feeding so had a gastrostomy tube placed.

He was enrolled on DOL 35, and a provisional diagnosis of Nemaline Myopathy was communicated on DOL 42. A diagnostic electromyogram and muscle biopsy under general anaesthesia were planned, but cancelled upon molecular diagnosis. He was discharged on DOL 45.

Molecular Diagnosis: Nemaline myopathy 2, *NEB* compound Heterozygous splice site Mutations - C → T c.19626+1G>A; C → G c.2416-1G>C

**Delphi round 2 are the following reasonable questions?**

Having a molecular diagnosis avoid the need for muscle biopsy?

Strongly disagree, Disagree, Neutral, Agree, Strongly agree, Unable to comment. Round 2 consensus: Agree.

Is it reasonable to use RCHSD data on the most recent neonate having a muscle biopsy for workup of hypotonia to estimate cost savings?

Strongly disagree, Disagree, Neutral, Agree, Strongly agree, Unable to comment. Round 2 consensus: Agree.

Given the clinical severity of this case and the fact that our control cases went to the ICU for 24-48 hours post muscle biopsy, is it reasonable to include such an ICU stay in the costs of avoided care?

Strongly disagree, Disagree, Neutral, Agree, Strongly agree, Unable to comment. Round 2 consensus: Agree.

**Statistical Analysis**

Comparisons of paired data used McNemar’s χ^2^ test. Fisher’s exact test was used to compare rates of occurrence.

**Supplemental Results**

rWGS was performed to an average depth of coverage of 46-fold (minimum 38-fold; Table S2). Completeness of rWGS was assessed by the proportion of 15,643 genes in Mendelian Inheritance in Man with >10-fold coverage of all coding domain nucleotides (median 97.9%)^16^.

**Patients with diagnoses that did not change management**

Four diagnoses did not change management. Infant 6002 had an inherited *SERPINA1* variant that partially explained cholestasis in an infant receiving appropriate treatment based on clinical diagnosis. Neonate 6004 had apnoea and seizures who had *de novo* isodicentric chromosome 15 syndrome, which was detected concurrently by CMA and rWGS, and subsequent changes in care were not attributed to rWGS. Infant 6028 had a *de novo RET* variant causative for Hirschprung disease, which did not change acute management. Infant 6029 had myelomeningocele and hydrocephalus and a paternally inherited *CELSR1* variant, which did not change acute management. Infant 6034 had anuria associated with multicystic dysplastic kidneys, prune belly and pulmonary hypoplasia. The diagnosis of 12q21.33q22DEL was made by CMA and rWGS after death (DOL 29). The diagnosis of adenovirus infection by metagenomic analysis of rWGS was not made prior to discharge, and consequently did not inform acute management. That infant presented with worsening seizures in the context of a known seizure disorder.

**Patients with acute changes in management who did not undergo healthcare utilization modelling**

In seven of thirteen infants, the impact of precision medicine could not be quantified without a group of matched historical controls and/or long-term follow up.

**Patient 6018** was compound heterozygous for two likely pathogenic variants in *POLR1C* (p.Leu81Pro and p.Arg109). *POLR1C* is associated autosomal recessive type 3 Treacher Collins syndrome and hypomyelinating leukodystrophy 11. This infant’s primary presentation was cardiomegaly and long QT syndrome. The diagnosis led to neurology consultation: the infant did not have evidence of type 3 Treacher Collins syndrome or hypomyelinating leukodystrophy 11. Treacher Collins syndrome has previously been associated with long QT syndrome^17^. Further follow up is warranted. There was no acute change in resource utilization related to the diagnosis.

**Patient 6019** had a causal *de novo* *GABRA1* variant and seizure disorder. The diagnosis confirmed that he was receiving the correct anti-epileptic regimen. He was readmitted shortly after the molecular diagnosis was made due to increased seizures in the context of an acute illness. Knowledge of the molecular diagnosis prevented empirical changes to the antiepileptic regimen. It was not possible to quantify the impact of a potential future change to an inferior antiepileptic regimen on healthcare utilization or outcome^6^.

**Patient 6020** presented with cardiomegaly, heart failure, left ventricular non-compaction, and cardiomyopathy. Diagnosis of left ventricular non-compaction type 9, associated with a *TPM1* variant, clarified that cardiac transplantation would be curative. While this diagnosis provided earlier clearance for cardiac transplant, precise determination of impact on healthcare utilization was not possible.

**Patient 6021** was a female infant with Early Infantile Epileptic Encephalopathy 9 associated with a paternally inherited *PCDH19* variant. A sibling received the same diagnosis. She was referred for a clinical trial of adjunct therapy with ganaxolone. However, the trial site was far from their home, her mother felt that he was adequately controlled on her current medication, and decided not to enrol her in the trial.

**Patient 6024** was a male infant with congenital heart disease and other congenital anomalies. He was found to have a *PHEX* variant that will lead to X-linked hypophosphatemic rickets. He had hypophosphatemia and vertebral anomalies that may be explained by his diagnosis. He was referred to endocrinology, and will be started on treatment prior to being symptomatic. While avoidance of rickets is likely to be associated with increased quality adjusted life years (QALYs), this was not possible to quantify.

**Patient 6030** had heart failure due to congenital heart disease (pulmonary valve atresia), and recurrent bacterial infections. He had a premature stop codon in *NF1*, indicating a diagnosis of Neurofibromatosis type 1. *NF1* is associated with congenital heart disease, including pulmonary atresia^18-21^. Given this diagnosis, he was evaluated by ophthalmology, neurology and nephrology, and had surveillance magnetic resonance imaging of his brain and renal arteries. Early diagnosis may result in early detection of *NF1*-associated tumours, but it was not possible to model the effect of early diagnosis on outcome or resource utilization.

**Patient 6056** was diagnosed with megacystic microcolon intestinal hypoperistalsis syndrome. He was enrolled on a clinical trial of cisaspride as a prokinetic agent^22^. He has had marked improvement in gut motility, and may avoid liver and intestinal transplant^23^.

It has been estimated that an average ~$500,000 in hospital charges would be impacted if paediatric palliative care was focused on infants with 50% percent predicted probability of dying during that hospitalization^24^.

**Table S1: Human Phenotype Ontology (HPO) terms for clinical features in the forty two proband inpatient infants. The rank and P-value for the causative gene are derived from Phenomizer^S7,S8^.**

| **Patient ID** | **HPO #** | **Human Phenotype Ontology Term** | **Primary Organ System Involved** | **Causative Gene** | **Rank** | **P-value** |
| --- | --- | --- | --- | --- | --- | --- |
| 6001 | HP:0001684  HP:0001331  HP:0030283  HP:0007370  HP:0001338  HP:0011451  HP:0001276  HP:0006895  HP:0200049  HP:0100490  HP:0008619  HP:0008513  HP:0000486  HP:0000487  HP:0004626  HP:0002944  HP:0004626  HP:0000921  HP:0001385  HP:0005407  HP:0001511  HP:0000519 | Atrial septal defect  Absence cavum septum pellucidum, complete  Absence cavum septum pellucidum, partial  Corpus callosum hypoplasia/partial absence  Callosal agenesis or severe hypogenesis  Congenital microcephaly  Hypertonia  Hypertonia, lower limbs  Hypertonia, upper limbs  Camplodactyly  Bilateral hearing loss, sensorineural  Bilateral hearing loss, conductive  Strabismus  Congenital strabismus  Scoliosis  Thoracolumbar scoliosis  Lumbar scoliosis  Missing ribs  Bilateral hip dysplasia, congenital  Low CD4+ T cell subset  In utero growth restriction  Congenital cataracts | Multiple Congenital Anomalies |  |  |  |
| 6002 | HP:0002908  HP:0008282  HP:0002910  HP:0003155  HP:0000952 | Conjugated hyperbilirubinemia  Unconjugated hyperbilirubinemia  Elevated hepatic transaminases  Elevated alkaline phosphatase  Jaundice | Liver | *SERPINA1*  (het) | 483 | 0.34 |
| 6003 | HP:0012469  HP:0001250 | Infantile spasms  Seizures | Neurologic |  |  |  |
| 6004 | HP:0002870  HP:0002871 | Obstructive sleep apnoea  Central sleep apnoea | Neurologic | tetrasomy 15q1.2q13.1 |  |  |
| 6005 | HP:0001903  HP:0003265 | Anaemia  Neonatal hyperbilirubinemia | Liver |  |  |  |
| 6009 | HP:0001511  HP:0005160  HP:0011682  HP:0001684  HP:0001171  HP:0006101  HP:0000308  HP:0000347  HP:0000219  HP:0008583  HP:0001894  HP:0012408 | In utero growth restriction  Total anomalous pulmonary venous return  Perimembranous ventricular septal defect  Moderate atrial septal defect  Ectrodactyly of the right hand  Syndactyly  Microretrognathia  Micrognathia  Thin protruding upper lip  Underfolded superior helices  Thrombocytosis  Medullary nephrocalcinosis | Multiple Congenital Anomalies |  |  |  |
| 6011 | HP:0002240  HP:0001744  HP:0002908  HP:0000952  HP:0002910  HP:0003155  HP:0006254  HP:0200084 | Hepatomegaly  Splenomegaly  Conjugated hyperbilirubinemia  Jaundice  Elevated hepatic transaminases  Elevated alkaline phosphatase  Elevated alpha-feto-protein  Giant cell hepatitis | Liver | *NPC1* | 327 | 0.015 |
| 6012 | HP:0001511  HP:000776  HP:0030680  HP:0002079  HP:0001999  HP:0002205  HP:0002194 | Small for gestational age  Congenital diaphragmatic hernia  Congenital heart disease  Hypoplasia of corpus callosum  Abnormal face shape  Recurrent respiratory infections  Delayed gross motor development | Multiple Congenital Anomalies | *ARID1B* | 283 | 0.74 |
| 6014 | HP:0001290  HP:0002835  HP:0000347  HP:0000463 | Generalized hypotonia  Aspiration  Micrognathia  Anteverted nares | Musculoskeletal | *NEB* | 654 | 1 |
| 6017 | HP:0005242  HP:0002908  HP:0002910  HP:0003128 | Extrahepatic biliary duct atresia  Conjugated hyperbilirubinemia  Elevated transaminases  Lactic acidosis | Liver |  |  |  |
| 6018 | HP:0001657  HP:0005184  HP:0002092  HP:0001385  HP:0011451  HP:0011968  HP:0002835  HP:0001508 | Prolonged QT syndrome  Prolonged QTc syndrome  Pulmonary hypertension  Hip dysplasia  Congenital microcephaly  Feeding difficulties  Aspiration  Failure to Thrive | Multiple Congenital Anomalies | *POLR1C* | 1695 | 1 |
| 6019 | HP:0012469  HP:0001250  HP:0002376  HP:0000565  HP:0008947 | Infantile spasms  Seizures  Developmental regression  Esotropia  Infantile muscular hypotonia | Neurologic | *GABRA1* | 4 | 0.016 |
| 6020 | HP:0001789  HP:0001791  HP:0001698  HP:0001711  HP:0030682  HP:0001646  HP:0012304  HP:0011649  HP:0001708  HP:0009937  HP:0000494  HP:0011247  HP:0010946  HP:0004719  HP:0001873  HP:0001942 | Hydrops fetalis  Foetal ascites  Pericardial effusion  Left ventricular abnormality  Left ventricular noncompaction  Abnormal aortic valve  Hypoplastic aortic arch  Patent ductus arteriosus after premature birth  Right ventricular failure  Facial hirsutism  Downslanting palpebral fissures  Prominent antihelix, crimped superiorly  Dilation of the renal pelvis  Hyperechogenic kidneys  Thrombocytopenia  Metabolic acidosis | Multiple Congenital Anomalies | *TPM1* | 21 | 0.0003 |
| 6021 | HP:0002104  HP:0001250 | Apnea  Seizures | Neurologic | *PCDH19* | 154 | 0.99 |
| 6022 | HP:0002104  HP:0005949  HP:0002791  HP:0012416  HP:0001601  HP:0005483  HP:3000053  HP:0011415  HP:0000347  HP:0004209  HP:0001511 | Apnea  Apnoeic episodes in infancy  Hypoventilation  Hypercapnia  Laryngomalacia  Abnormality of the epiglottis  Abnormality of the hypopharynx  Calcified placenta  Micrognathia  Clinodactyly  In utero growth restriction | Multiple Congenital Anomalies |  |  |  |
| 6023 | HP:0011968  HP:0002878  HP:0002133  HP:0001250 | Feeding difficulties  Respiratory failure  Status epilepticus  Seizures | Neurologic |  |  |  |
| 6024 | HP:0001680  HP:0012304  HP:0011682  HP:0011670  HP:0011668  HP:0001655  HP:0000062  HP:0000033  HP:0000808  HP:0000041  HP:0000960  HP:0003468  HP:0006380  HP:0001511  HP:0000347  HP:0001873  HP:0001905 | Coarctation of the aorta  Hypoplastic aortic arch  Perimembranous VSD  Left superior vena cava to coronary sinus  Bilateral superior vena cava  Patent foramen ovale  Ambiguous external genitalia at birth  Ambiguous genitalia, male  Penoscrotal hypospadias  Chordee  Sacral dimple  Abnormality of the vertebrae  Knee flexion contractures  In utero growth restriction  Mild micrognathia  Thrombocytopenia  Congenital thrombocytopenia | Multiple Congenital Anomalies | *PHEX* | 2792 | 1 |
| 6026 | HP:0002908  HP:0001396  HP:0004971  HP:0001643  HP:0001942  HP:0001947  HP:0001508 | Conjugated hyperbilirubinemia  Cholestasis  Pulmonary artery hypoplasia  Patent ductus arteriosus  Metabolic acidosis  Renal tubular acidosis  Failure to thrive | Liver | *JAG1 (3Mb deletion)* | 72 | 0.30 |
| 6027 | HP:0004430  HP:0005407  HP:0005352  HP:0000776  HP:0003645  HP:0010972 | Severe combined immune deficiency  Decreased number of CD4 cells  Severe T cell immunodeficiency  Congenital diaphragmatic hernia  Prolonged partial thromboplastin time  Anaemia due to inadequate production | Hematologic |  |  |  |
| 6028 | HP:0005241  HP:0011286  HP:0011464 | Total intestinal aganglionosis  Total colonic aganglionosis  Aganglionosis of the small intestine | Gastrointestinal | *RET* | 8 | 0.0003 |
| 6029 | HP:0002475  HP:0000238  HP:0030048  HP:0007370  HP:0002308 | Myelomeningocele  Congenital hydrocephalus  Colpocephaly  Congenital absence of corpus callosum  Arnold Chiari Malformation Type II | Neurologic | *CELSR1* | n/a | n/a |
| 6030 | HP:0030680  HP:0004935  HP:0000967  HP:0000938  HP:0002788  HP:0002742  HP:0002726 | Congenital heart disease  Pulmonary valve atresia  Petechiae  Osteopenia  Recurrent upper respiratory infections  Recurrent Klebsiella infections  Recurrent Staphylococcus infections | Multiple Congenital Anomalies | *NF1* | 2735 | 1 |
| 6032 | HP:0010959  HP:0001888 | Congenital cystic adenomatoid malformation of lung  Lymphopenia | Pulmonary |  |  |  |
| 6034 | HP:0000003  HP:0004392  HP:0002089  HP:0000028  HP:0100519  HP:0002153  HP:0002905  HP:0002901 | Multicystic dysplastic kidney  Prune belly  Pulmonary hypoplasia  Cryptorchidism  Anuria  Hyperkalemia  Hyperphosphatemia  Hypocalcaemia | Multiple Congenital Anomalies | 12q21.33q22DEL | n/a | n/a |
| 6036 | HP:0001250  HP:0001287  HP:0002383  HP:0007105  HP:0012747  HP:0012229  HP:0012756  HP:0002922  HP:0002353 | Seizures  Meningitis  Encephalitis  Infantile encephalopathy  Abnormal brain MRI  CSF pleocytosis  CSF polymorphonuclear pleocytosis  Elevated CSF protein  Abnormal EEG | Neurologic |  |  |  |
| 6037 | HP:0002908  HP:0005242  HP:0002910  HP:0008282  HP:0000952 | Conjugated hyperbilirubinemia  Extrahepatic biliary duct atresia  Elevated transaminases  Unconjugated hyperbilirubinemia  Jaundice | Liver |  |  |  |
| 6038 | HP:0011863  HP:0001539  HP:0011623  HP:0001684  HP:0004927  HP:0000776  HP:0002092  HP:0002089  HP:0002878  HP:0001334  HP:0007165  HP:0010946 | Sternal ossification center anomalies  Omphalocele  Moderate/large mid muscular VSD  Moderate/large secundum atrial septal defect  Dilated main pulmonary artery  Left posterior congenital diaphragmatic hernia  Pulmonary hypertension  Pulmonary hypoplasia  Respiratory failure  Communicating hydrocephalus  Periventricular heterotopia  Renal pelviectasis | Multiple Congenital Anomalies |  |  |  |
| 6040 | HP:0002573  HP:0001824  HP:0011968  HP:0011102  HP:0002583 | Hematochezia  Weight loss  Feeding difficulties  Ileal atresia  Colitis | Gastrointestinal |  |  |  |
| 6041 | HP:0200134  HP:0010851  HP:0002123  HP:0011451 | Epileptic encephalopathy  EEG with burst suppression  Myoclonic epilepsy  Congenital microcephaly | Neurologic | *KCNQ2* | 2 | 0.001 |
| 6046 | HP:0001695  HP:0005184  HP:0002153  HP:0001942  HP:0001987 | Cardiac arrest  Prolonged QTc interval  Hyperkalemia  Metabolic acidosis  Hyperammonemia | Cardiac |  |  |  |
| 6047 | HP:0005298  HP:0011579  HP:0004935  HP:0011563  HP:0011670  HP:0011649  HP:0011701  HP:0002104  HP:0006528  HP:0005100  HP:0001508  HP:0008872 | AVC with right ventricle aorta and pulmonary atresia  Unbalanced AV canal defect  Pulmonary atresia  Ventriculoatreial discordance  Left superior vena cava to coronary sinus  PDA after premature birth  Atrial tachycardia  Apnea  Chronic lung disease  Premature birth following PROM  Failure to thrive  Feeding difficulties in infancy | Cardiac |  |  |  |
| 6049 | HP:0010882  HP:0011612  HP:0011604  HP:0001643  HP:0001707  HP:0001714  HP:0004762  HP:0006704  HP:0001647  HP:0011726  HP:0008757  HP:0012821  HP:0001601  HP:0002625  HP:0001511  HP:0001508  HP:0002718  HP:0005420  HP:0000260  HP:0005556  HP:0005280  HP:0002056  HP:0001076  HP:0000463 | Pulmonary valve atresia  Type A interruption of the pulmonary artery  Large aorto-pulmonary window  Large patent ductus arteriosus  Abnormality of the right ventricle  Ventricular hypertrophy  Severe right ventricular hypoplasia  Right ventricular sinusoids  Bicuspid aortic valve  Persistent fetal circulation  Unilateral vocal cord paralysis  Unilateral vocal cord paresis  Laryngomalacia with redundant arytenoids prolapsing into airway  Deep venous thrombosis  In utero growth restriction  Poor weight gain  Multiple bacterial infections  Multiple bacterial infections with gram negative organisms  Large anterior fontanel  Delayed metopic suture closure  Very depressed nasal bridge  Furrows in glabellar region with flat vascular malformation  Glabbelar capillary hemangioma  Anteverted nares | Cardiac |  |  |  |
| 6053 | HP:0000842  HP:0001943  HP:0001520  HP:0200128  HP:0002643 | Hyperinsulinemia  Hypoglycemia  Large for gestational age  Biventricular hypertrophy  Neonatal respiratory distress | Endocrine/Biochemical | *ABCC8* | 2 | 0.004 |
| 6056 | HP:0000021  HP:0004388  HP:0100771  HP:0002566  HP:0002144  HP:0000072 | Megacystis  Microcolon  Hypoperistalsis  Intestinal malrotation  Tethered cord  Hydroureter | Gastrointestinal | *ACTG2* | 4 | 0.0015 |
| 6060 | HP:0002013  HP:0000822  HP:0001946  HP:0001508 | Vomiting  Hypertension  Ketosis  Failure to thrive | Endocrine/Biochemical |  |  |  |
| 6064 | HP:0030853  HP:0001748  HP:0011578  HP:0010445  HP:0001680  HP:0001659  HP:0011103  HP:0011589  HP:0011671  HP:0011669  HP:0004794  HP:0000121 | Heterotaxy syndrome  Polysplenia  Atrioventricular canal, transitional  Large primum atrial septal defect  Coarctation  Mild aortic valve regurgitation  Abnormality of the left ventricular outflow tract  Left aortic arch with common brachiocephalic trunk  Interrupted inferior vena cava with azygous connection to the right superior vena cava  Let superior vena cava directly joins left atrium  Small bowel malrotation  Nephrocalcinosis | Multiple Congenital Anomalies |  |  |  |
| 6065 | HP:0001680  HP:0011622  HP:0011625  HP:0001643  HP:0011667  HP:0001274  HP:0012762  HP:0030048  HP:0002418  HP:0001321  HP:0001320  HP:0006951  HP:0000175  HP:0000126  HP:0012435 | Severe coarctation of the aorta  Large inlet ventricular septal defect  Multiple muscular ventricular septal defects  Large patent ductus arteriosus  Bilateral superior vena cava with small bridging vein  Agenesis of the corpus callosum  Cerebral white matter atrophy  Colpocephaly  Abnormality of midbrain morphology  Hypoplastic cerebellar hemispheres  Hypoplastic cerebellar vermis  Prominent retrocerebellar cyst  Cleft palate  Hydronephrosis  Ventral shortening of foreskin | Multiple Congenital Anomalies |  |  |  |
| 6066 | HP:0002908  HP:0002910  HP:0002744  HP:0001721 | Conjugated hyperbilirubinemia  Elevated transaminases  Bilateral cleft lip and palate  Abnormal hair whorl | Liver |  |  |  |
| 6069 | HP:0002908  HP:0002910  HP:0001396 | Direct hyperbilirubinemia  Elevated hepatic transaminases  Cholestasis | Liver |  |  |  |
| 6070 | HP:0001250  HP:0001297  HP:0005543  HP:0000952  HP:0003265  HP:0001935  HP:0001976 | Seizures  Stroke  Protein C Deficiency  Hyperbilirubinemia  Jaundice  Microcytic anemia  Anti-thrombin III low | Neurologic |  |  |  |
| 6071 | HP:0003256  HP:0001876  HP:0002019  HP:0011968 | Abnormality of the clotting cascade  Pancytopenia  Constipation  Feeding difficulties | Hematologic |  |  |  |
| 6072 | HP:0004812  HP:0002715  HP:0002841 | Pre-B cell acute lymphoblastic leukemia  Abnormality of the immune system  Recurrent fungal infection | Hematologic |  |  |  |

**Table S2: Metrics of rapid whole genome sequencing for forty two proband inpatient infants.** Abbreviations. GB: gigabase; nt: nucleotides; MIM: Mendelian inheritance in Man; CD: coding domain; indels: insertion-deletion nucleotide variants; SNVs: single nucleotide variants; Hom: homozygous; Het: heterozygous; Ti: nucleotide transition; Tv: nucleotide transversion; *: run on HiSeq 2500 in rapid run mode.

| **Family ID** | **Raw sequence (GB)** | **% reads mapped** | **% duplicates** | **Yield (GB)** | **Mean Insert size (nt)** | **Average coverage** | **MIM genes with <10X coverage at 100% CD nt** | **MIM genes with >10X coverage at 100% of CD nt** | **Nt variant calls** | **Passing nt variant calls** | **CD nt variants** | **SNVs** | **Indels** | **Nt variant Hom /Het ratio (in CD)** | **Ti/Tv ratio (in CD)** |
| --- | --- | --- | --- | --- | --- | --- | --- | --- | --- | --- | --- | --- | --- | --- | --- |
| 6001 | 190.6 | 99.0% | 11.8% | 166.5 | 332.2 | 50.0 | 276 | 98.0% | 5060268 | 4881770 | 27452 | 4050165 | 889492 | 0.56 (0.59) | 2.03 (2.93) |
| 6002 | 191.7 | 99.0% | 13.3% | 164.6 | 345.3 | 49.0 | 258 | 98.2% | 5031505 | 4858836 | 27027 | 4032862 | 881511 | 0.58 (0.62) | 2.03 (2.84) |
| 6003 | 188.9 | 99.0% | 16.4% | 156.4 | 345.2 | 48.0 | 261 | 98.2% | 4893857 | 4732048 | 27452 | 3922074 | 861711 | 0.59 (0.58) | 2.04 (2.88) |
| 6004 | 157.4 | 99.1% | 14.2% | 133.6 | 348.7 | 41.5 | 258 | 98.2% | 4888733 | 4754885 | 26368 | 3949520 | 872691 | 0.59 (0.59) | 2.04 (2.97) |
| 6005 | 198.2 | 99.2% | 19.8% | 157.4 | 330.3 | 47.0 | 255 | 98.2% | 4827128 | 4760085 | 26676 | 3954474 | 872654 | 0.58 (0.57) | 2.04 (2.92) |
| 6009 | 163.1 | 98.7% | 10.4% | 144.8 | 341.8 | 43.0 | 316 | 97.8% | 4788941 | 4702331 | 26445 | 3934041 | 854900 | 0.57 (0.58) | 2.04 (2.95) |
| 6011 | 195.8 | 98.9% | 12.1% | 170.4 | 352.4 | 51.0 | 254 | 98.2% | 4613310 | 4539914 | 25211 | 3773668 | 839642 | 0.84 (0.97) | 2.03 (2.93) |
| 6012 | 195.5 | 98.8% | 10.0% | 174.1 | 369.3 | 52.0 | 254 | 98.2% | 4945287 | 4867107 | 27343 | 4059083 | 886204 | 0.53 (0.56) | 2.04 (2.97) |
| 6014 | 183.8 | 98.7% | 10.3% | 163.2 | 375.6 | 49.0 | 279 | 98.0% | 4912310 | 4859086 | 27238 | 4027916 | 884394 | 0.55 (0.58) | 2.04 (2.92) |
| 6017 | 199.2 | 98.9% | 15.3% | 190.4 | 351.0 | 49.2 | 288 | 98.0% | 5028028 | 4915951 | 26917 | 4038370 | 877581 | 0.56 | 1.98 |
| 6018 | 197.8 | 98.6% | 10.0% | 179.5 | 392.4 | 49.3 | 285 | 98.0% | 5083464 | 4975587 | 25953 | 4088583 | 887004 | 0.49 | 1.98 |
| 6019 | 176.2 | 99.0% | 11.2% | 154.9 | 361.8 | 46.0 | 268 | 98.1% | 4853103 | 4760785 | 26917 | 3982176 | 870927 | 0.58 | 2.04 |
| 6020* | 144.9 | 98.4% | 4.1% | 137.5 | 407.6 | 42.0 | 520 | 96.3% | 4787333 | 4685966 | 26064 | 3913210 | 874123 | 0.67 | 2.03 |
| 6021 | 190.0 | 99.1% | 11.3% | 166.8 | 351.4 | 45.3 | 324 | 97.7% | 5042004 | 4937248 | 27247 | 4051442 | 885806 | 0.52 | 1.98 |
| 6022 | 198.8 | 98.8% | 10.6% | 175.9 | 367.7 | 47.8 | 325 | 97.7% | 5047008 | 4940229 | 27548 | 4058582 | 881647 | 0.52 | 1.98 |
| 6023 | 205.6 | 98.8% | 11.8% | 179.6 | 334.8 | 48.2 | 388 | 97.3% | 5003363 | 4893589 | 27217 | 4019302 | 874287 | 0.54 | 1.98 |
| 6024 | 217.8 | 98.8% | 14.9% | 183.5 | 358.3 | 47.6 | 315 | 97.8% | 4974795 | 4862566 | 26778 | 3996325 | 866241 | 0.55 | 1.98 |
| 6026* | 135.1 | 98.6% | 2.9% | 129.9 | 390.6 | 40.6 | 215 | 98.5% | 4763155 | 4720511 | 26645 | 3940960 | 876245 | 0.63 | 2.03 |
| 6027 | 223.7 | 99.0% | 12.9% | 192.9 | 336.9 | 51.3 | 302 | 97.9% | 5004293 | 4888107 | 27209 | 4012999 | 875108 | 0.58 | 1.98 |
| 6028 | 189.4 | 99.0% | 18.7% | 152.4 | 336.6 | 44.0 | 330 | 97.7% | 5496640 | 5498529 | 30981 | 4647142 | 1026126 | 0.39 | 2.04 |
| 6029 | 207.2 | 98.9% | 21.2% | 204.9 | 326.0 | 49.3 | 285 | 98.0% | 4938020 | 4812763 | 26804 | 3965375 | 847388 | 0.55 | 1.96 |
| 6030 | 275.0 | 98.9% | 25.7% | 202.0 | 351.9 | 45.8 | 268 | 98.1% | 4932826 | 4820090 | 26800 | 3956885 | 863205 | 0.68 | 1.96 |
| 6032 | 199.9 | 99.2% | 25.2% | 148.0 | 313.0 | 44.0 | 273 | 98.1% | 5092499 | 4973259 | 27438 | 4085534 | 887725 | 0.50 | 1.96 |
| 6034 | 193.2 | 99.0% | 15.0% | 162.6 | 328.4 | 49.0 | 339 | 97.6% | 4905722 | 4782179 | 26708 | 3924626 | 857553 | 0.58 | 1.96 |
| 6036 | 184.3 | 98.6% | 12.1% | 181.6 | 377.6 | 48.5 | 592 | 95.8% | 4978741 | 4871764 | 27174 | 4004764 | 867000 | 0.56 | 1.96 |
| 6037 | 173.5 | 98.8% | 8.7% | 171.5 | 334.4 | 47.8 | 380 | 97.3% | 4987816 | 4874388 | 27128 | 4008447 | 865941 | 0.57 | 2.02 |
| 6038 | 177.6 | 98.9% | 9.1% | 175.7 | 339.4 | 48.7 | 337 | 97.6% | 4909747 | 4794272 | 26569 | 3938653 | 855619 | 0.57 | 1.96 |
| 6040 | 179.1 | 98.9% | 13.5% | 177.2 | 407.3 | 46.5 | 262 | 98.1% | 4991038 | 4880382 | 27579 | 4042643 | 837739 | 0.59 | 1.92 |
| 6041 | 144.8 | 98.8% | 11.9% | 143.1 | 412.7 | 38.5 | 294 | 97.9% | 4709835 | 4626335 | 25822 | 3835022 | 791313 | 0.82 | 1.93 |
| 6046 | 168.6 | 98.5% | 9.7% | 166.4 | 408.3 | 45.8 | 268 | 98.1% | 4977590 | 4891090 | 26856 | 4016048 | 875042 | 0.54 | 1.95 |
| 6047 | 169.0 | 98.2% | 14.4% | 156.0 | 417.0 | 43.0 | 325 | 97.7% | 5111922 | 5026580 | 27903 | 4127940 | 898640 | 0.58 | 1.96 |
| 6049 | 155.7 | 98.8% | 7.6% | 153.8 | 395.8 | 42.1 | 445 | 96.8% | 4975793 | 4885594 | 27524 | 4030736 | 854858 | 0.58 | 1.94 |
| 6053 | 163.0 | 99.0% | 9.1% | 161.3 | 413.1 | 44.7 | 382 | 97.3% | 4911276 | 4800903 | 27304 | 3978163 | 822740 | 0.66 | 1.96 |
| 6056 | 152.2 | 99.0% | 11.0% | 150.8 | 389.4 | 41.0 | 287 | 98.0% | 5062331 | 4961009 | 27716 | 4112836 | 848173 | 0.54 | 1.93 |
| 6060 | 144.0 | 99.0% | 6.8% | 142.5 | 411.6 | 40.5 | 264 | 98.1% | 5357265 | 5241544 | 29458 | 4360768 | 880776 | 0.44 | 1.93 |
| 6064 | 202.7 | 98.9% | 8.2% | 200.6 | 411.5 | 56.1 | 239 | 98.3% | 5017220 | 4876361 | 27611 | 4034132 | 842229 | *0.64* | 1.91 |
| 6065 | 164.5 | 98.9% | 8.6% | 162.6 | 414.7 | 45.2 | 348 | 97.5% | 4966944 | 4829817 | 27275 | 4011528 | 818289 | 0.56 | 1.90 |
| 6066* | 149.3 | 98.6% | 2.9% | 147.2 | 415.5 | 43.6 | 333 | 97.6% | 4849444 | 4755483 | 26075 | 3880241 | 875242 | 0.75 | 1.95 |
| 6069* | 141.7 | 98.5% | 2.7% | 139.5 | 404.0 | 41.5 | 338 | 97.6% | 4998354 | 4923491 | 27045 | 4040738 | 882753 | 0.61 | 1.97 |
| 6070* | 160.8 | 96.4% | 2.8% | 155.0 | 390.0 | 40.5 | 363 | 97.4% | 4948313 | 4872395 | 26859 | 3997776 | 874619 | 0.57 | 1.96 |
| 6071* | 133.4 | 98.6% | 2.8% | 131.4 | 385.7 | 39.1 | 379 | 97.3% | 4985721 | 4894714 | 26499 | 4004354 | 890360 | 0.58 | 1.95 |
| 6072* | 131.7 | 98.4% | 2.9% | 129.7 | 393.3 | 38.4 | 698 | 95.1% | 4999653 | 4906812 | 26826 | 4017442 | 889370 | 0.59 | 1.95 |
| **Average** | 178.9 | 98.8% | 11.3% | 162.8 | 370.7 | 45.8 | 325 | 97.7% | 4967919 | 4865151 | 27087 | 4019703 | 870592 | 0.58 | 1.97 |
| **Minimum** | 131.7 | 96.4% | 2.7% | 129.7 | 313.0 | 38.4 | 215 | 95.1% | 4613310 | 4539914 | 25211 | 3773668 | 791313 | 0.39 | 1.90 |
| **Maximum** | 275 | 99.2% | 25.7% | 204.9 | 417.0 | 56.1 | 698 | 98.5% | 5496640 | 5498529 | 30981 | 4647142 | 1026126 | 0.82 | 2.04 |
| **Median** | 181.45 | 98.9% | 11.1% | 162.6 | 368.5 | 45.9 | 298 | 97.9% | 4976692 | 4872080 | 27036 | 4012264 | 874205 | 0.57 | 1.96 |

**Table S3: Presentation and inheritance pattern in eighteen infants who were diagnosed (Dx) with genetic disorders.**

| **Subject ID** | **Dx Type** | **Mode of Dx** | **Primary Clinical Features** | **Diagnosis Name(s)** | **Gene(s)** | **Inheritance Pattern(s)** | ***de novo* or inherited** | **Variant Chromosomal (Chr) or Gene (c.) Coordinate(s)** | **Variant Protein Coordinate(s)** | **ACMG**  **Classification** |  |
| --- | --- | --- | --- | --- | --- | --- | --- | --- | --- | --- | --- |
| 6002 | P | rWGS | Cholestasis | alpha-1-antitrypsin "Z" heterozygous state | *SERPINA1* | AR | Inherited  (Paternal) | chr14:94844947  c.1096G>A | p.Glu366Lys | Pathogenic |  |
| 6004 | C | CMA rWGS | Apnoea  Seizures | Isodicentric chromosome 15 syndrome | Multiple | AD | *de novo* | 15q11.2q13.1  (22,770,421-30,386,552)x4,  15q13.1q13.3  (30,913,573-32,428,067)x3 | *NA* | Pathogenic |  |
| 6011 | C | rWGS | Cholestasis | Niemann-Pick disease type C | *NPC1* | AR | Inherited | chr18:21119857  c.2713C>T | p.Gln905Ter | Likely pathogenic |  |
| 6012 | C | rWGS | Congenital Diaphragmatic Hernia and Heart Disease  Frequent Infections | Coffin-Siris Syndrome | *ARID1B* | AD | *de novo* | chr6:157495210  c.3096_3100delCAAAG | p.Lys1033ArgfsTer32 | Likely Pathogenic |  |
| 6014 | C | rWGS | Hypotonia | Nemaline myopathy | *NEB* | AR | n.d. (duo)  Inherited (maternal) | chr2:152410341  c.19626+1G>A  chr2:152544248  c.2416-1G>C | canonical splice donor  canonical splice acceptor | Likely pathogenic  Likely pathogenic |  |
| 6018 | P | rWGS | Long QT syndrome  Cardiomegaly | Treacher Collins  syndrome (TCS)/ hypomyelinating leukodystrophy 11 | *POLR1C* | AR | Inherited  (maternal)  Inherited  (paternal) | chr6:43487171  c.242T>C  chr6:43487520  c.326G>A | p.Leu81Pro  p.Arg109His | Likely pathogenic  Likely pathogenic |  |
| 6019 | C | rWGS | Seizures | Epileptic encephalopathy, Early infantile, type 19 | *GABRA1* | AD | *de novo* | chr5:161317989  c.789G>A | p.Met263Ile | Likely pathogenic |  |
| 6020 | C | rWGS | Cardiomyopathy  Heart Failure | Left ventricular non-compaction 9 | *TPM1* | AD | n.d. (duo) | chr15:63353108  c.533G>A | p.Arg178His | Likely pathogenic |  |
| 6021 | C | rWGS | Seizures | Epileptic encephalopathy, Early infantile, type 9 | *PCDH19* | AD | Inherited (paternal) | chrX:99662806  c.790G>T | p.Asp264Tyr | Likely pathogenic |  |
| 6024 | P | rWGS | Congenital Heart Defect  Hypophosphatemia | X-linked hypophosphatemia rickets | *PHEX* | XLD | Inherited (maternal) | chrX:22208578  c.1604C>T | p.Thr535Met | Likely pathogenic |  |
| 6026 | C | rWGS CMA | Cholestasis | Alagille Syndrome | *JAG1* | AD | *de novo* | chr20:10,471400-13,459,333  3MB heterozygous deletion | *NA* | pathogenic |  |
| 6028 | C | rWGS | Ileal Stenosis, Microcolon  Total intestinal aganglionosis | Hirschsprung Disease | *RET* | AD | *de novo* | chr10:43619168  c.2851C>T | p.Pro951Ser | Likely pathogenic |  |
| 6029 | C | rWGS | Myelomeningocele  Hydrocephalus | Neural Tube Defect (Spina Bifida) | *CELSR1* | AD | Inherited  (Paternal) | chr22:46931538  c.1529dupA | p.His510GlnfsTer26 | Likely pathogenic |  |
| 6030 | C | rWGS | Congenital Heart Defect  Heart Failure  Frequent Fevers  Petechiae | Neurofibromatosis Type 1  Cardiomyopathy | *NF1*  *MYBPC3* | AD  AD | *de novo*  Inherited  (maternal) | chr17:29653118  c.5118delT  chr11:47355113  c.3184delG | p.Val1707PhefsTer3  p.Val1062LeufsTer13 | Likely pathogenic  Likely pathogenic |  |
| 6034 | C | rWGS CMA | Prune belly, mullticystic dysplastic kidney, pulmonary hypoplasia, cryptorchidism | 3.6 Mb loss of 12q21.33-q22 | Multiple | AD | *de novo* | 12q21.33q22  (91,454,789-95,021,920)x1 | *NA* | Likely pathogenic |  |
| 6041 | C | rWGS | Seizures | Epileptic Encephalopathy, Early Infantile, type 7 | *KCNQ2* | AD | *de novo* | chr20:62071003  c.875T>C | p.Leu292Pro | Likely pathogenic |  |
| 6053 | C | rWGS | Hypoglycaemia | Congenital hyperinsulinism | *ABCC8* | AD | Inherited (paternal) | chr11:17434263  c.2506C>T | p.Arg836Ter | pathogenic |  |
| 6056 | C | rWGS | Severe Microcolon  Malrotation | Megacystic  microcolon intestinal hypoperistalsis syndrome | *ACTG2* | AD | *de novo* | chr2:74140753  c.593G>A | p.Gly198Asp | Likely pathogenic |  |

C: Complete diagnosis; P: Partial diagnosis; CMA: Chromosomal microarray; AD: autosomal dominant; AR: autosomal recessive; XLD: X-linked dominant.

**Table S4: Analysis of cost of rWGS.** Included were consultation for pretest assessment, counselling, result disclosure, precision medicine guidance, and trio Sanger sequencing to validate variants detected by rWGS.

| **Direct Costs** | |  |  |  |  |  |  |  |
| --- | --- | --- | --- | --- | --- | --- | --- | --- |
|  |  |  |  |  |  |  |  |  |
| Labour |  | Singleton |  | Trio |  | Duo |  | Quad |
|  | Enrolment/Intake |  |  |  |  |  |  |  |
|  | Fellow | $31 |  | $31 |  | $31 |  | $31 |
|  | MD | $154 |  | $154 |  | $154 |  | $154 |
|  | Genomics Employees | $2,186 |  | $3,279 |  | $2,733 |  | $3,826 |
|  | Total Labour | $2,371 |  | $3,464 |  | $2,918 |  | $4,011 |
|  |  |  |  |  |  |  |  |  |
| Non-Labour | |  |  |  |  |  |  |  |
|  | IT | $604 |  | $1,813 |  | $1,208 |  | $2,417 |
|  | Sequencing | $2,898 |  | $8,694 |  | $5,796 |  | $11,592 |
|  | Data Analysis | $386 |  | $1,159 |  | $773 |  | $1,546 |
|  | Reporting | $150 |  | $150 |  | $150 |  | $150 |
|  | Total Non-Labour | $4,039 |  | $11,816 |  | $7,927 |  | $15,704 |
|  |  |  |  |  |  |  |  |  |
| **Indirect Costs** | |  |  |  |  |  |  |  |
|  | Genomics Non-Lab Employees | $891 |  | $891 |  | $891 |  | $891 |
|  | Supplies | $42 |  | $42 |  | $42 |  | $42 |
|  | Professional fees | $1 |  | $1 |  | $1 |  | $1 |
|  | Purchased services | $298 |  | $298 |  | $298 |  | $298 |
|  | Leases and rentals | $287 |  | $287 |  | $287 |  | $287 |
|  | Depreciation | $454 |  | $680 |  | $567 |  | $794 |
|  | Other expenses | $100 |  | $100 |  | $100 |  | $100 |
|  | Total Indirect Costs | $2,072 |  | $2,299 |  | $2,186 |  | $2,412 |
|  |  |  |  |  |  |  |  |  |
| **Total Cost per Infant** | | $8,482 |  | $17,579 |  | $13,031 |  | $22,128 |
|  |  |  |  |  |  |  |  |  |
| **Total Cost for 42 enrolled families** | | $674,645 |  |  |  |  |  |  |
| (3 singletons, 29 trios, 9 duos, 1 quad) | |  |  |  |  |  |  |  |

**Table S5: Time (days) from birth and admission to enrolment (consent), blood sample receipt, provisional diagnosis, final diagnosis, mortality, and final data extraction.**

**Table S6: Standard genetic tests ordered, and those which yielded a molecular diagnosis, and those which had acute clinical utility.** Abbreviations: rWGS: rapid whole genome sequencing; Std: standard; Mol: molecular; Dx: diagnosis; Cyto: cytogenetic; Biochem: biochemical; CMA: chromosomal microarray; AA: serum amino acids; UOA: urine organic acids; α1AT: α1-antitrypsin phenotype; CSF: cerebrospinal fluid; ATIII: antithrombin III; AC:acylcarnitine panel; AFP: α-fetoprotein; FISH: chromosomal fluorescence in situ hybridization.

**Figure S1: Workflow and median times from inpatient infant nomination for rWGS to reporting of confirmed diagnosis and implementation of precision medicine interventions.**


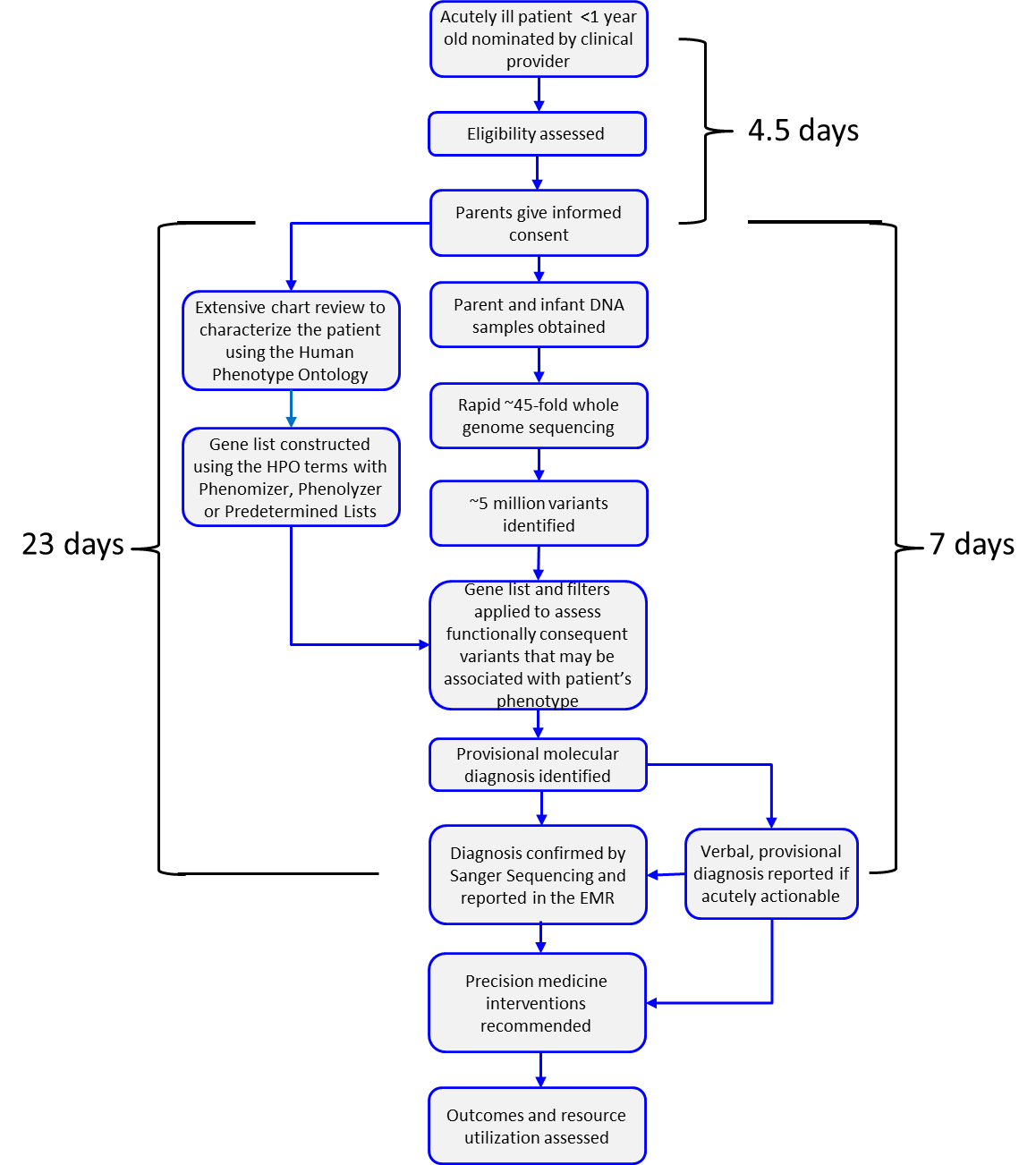


**Supplementary References**

1. Köhler S, Vasilevsky NA, Engelstad M, et al. The Human Phenotype Ontology in 2017. *Nucleic Acids Res.* 2016.

2. Yang H, Robinson PN, Wang K. Phenolyzer: phenotype-based prioritization of candidate genes for human diseases. *Nat Methods.* 2015;12(9):841-843.

3. Petrikin JE, Willig LK, Smith LD, Kingsmore SF. Rapid whole genome sequencing and precision neonatology. *Semin Perinatol.* 2015;39(8):623-631.

4. Soden SE, Saunders CJ, Willig LK, et al. Effectiveness of exome and genome sequencing guided by acuity of illness for diagnosis of neurodevelopmental disorders. *Sci Transl Med.* 2014;6(265):265ra168.

5. Hildreth A, Wigby K, Chowdhury S, et al. Rapid whole-genome sequencing identifies a novel homozygous NPC1 variant associated with Niemann-Pick type C1 disease in a 7-week-old male with cholestasis. *Cold Spring Harb Mol Case Stud.* 2017;3(5).

6. Farnaes L, Nahas SA, Chowdhury S, et al. Rapid whole-genome sequencing identifies a novel GABRA1 variant associated with West syndrome. *Cold Spring Harb Mol Case Stud.* 2017;3(5).

7. Pisano T, Numis AL, Heavin SB, et al. Early and effective treatment of KCNQ2 encephalopathy. *Epilepsia.* 2015;56(5):685-691.

8. Emerick KM, Rand EB, Goldmuntz E, Krantz ID, Spinner NB, Piccoli DA. Features of Alagille syndrome in 92 patients: frequency and relation to prognosis. *Hepatology.* 1999;29(3):822-829.

9. Lee HP, Kang B, Choi SY, Lee S, Lee SK, Choe YH. Outcome of Alagille Syndrome Patients Who Had Previously Received Kasai Operation during Infancy: A Single Center Study. *Pediatr Gastroenterol Hepatol Nutr.* 2015;18(3):175-179.

10. Kaye AJ, Rand EB, Munoz PS, Spinner NB, Flake AW, Kamath BM. Effect of Kasai procedure on hepatic outcome in Alagille syndrome. *J Pediatr Gastroenterol Nutr.* 2010;51(3):319-321.

11. Stanley CA, Thornton PS, Ganguly A, et al. Preoperative evaluation of infants with focal or diffuse congenital hyperinsulinism by intravenous acute insulin response tests and selective pancreatic arterial calcium stimulation. *J Clin Endocrinol Metab.* 2004;89(1):288-296.

12. Hussain K, Blankenstein O, De Lonlay P, Christesen HT. Hyperinsulinaemic hypoglycaemia: biochemical basis and the importance of maintaining normoglycaemia during management. *Arch Dis Child.* 2007;92(7):568-570.

13. Menni F, de Lonlay P, Sevin C, et al. Neurologic outcomes of 90 neonates and infants with persistent hyperinsulinemic hypoglycemia. *Pediatrics.* 2001;107(3):476-479.

14. Lam V, Kain N, Joynt C, van Manen MA. A descriptive report of end-of-life care practices occurring in two neonatal intensive care units. *Palliat Med.* 2016;30(10):971-978.

15. Oberender F, Tibballs J. Withdrawal of life-support in paediatric intensive care--a study of time intervals between discussion, decision and death. *BMC Pediatr.* 2011;11:39.

16. OMIM Entry Statistics. <https://www.omim.org/statistics/entry>. Accessed August 30, 2017.

17. Djurhuus MS, Klitgaard NA, Jensen BM, Andersen PE, Schrøder HD. Multiple anomalies, hypokalaemic paralysis and partial symptomatic relief by terbutaline. *Acta Paediatr.* 1998;87(4):475-477.

18. Nguyen R, Mir TS, Kluwe L, et al. Cardiac characterization of 16 patients with large NF1 gene deletions. *Clin Genet.* 2013;84(4):344-349.

19. İncecik F, Hergüner Ö, Alınç Erdem S, Altunbaşak Ş. Neurofibromatosis type 1 and cardiac manifestations. *Turk Kardiyol Dern Ars.* 2015;43(8):714-716.

20. Friedman JM, Arbiser J, Epstein JA, et al. Cardiovascular disease in neurofibromatosis 1: report of the NF1 Cardiovascular Task Force. *Genet Med.* 2002;4(3):105-111.

21. Brannan CI, Perkins AS, Vogel KS, et al. Targeted disruption of the neurofibromatosis type-1 gene leads to developmental abnormalities in heart and various neural crest-derived tissues. *Genes Dev.* 1994;8(9):1019-1029.

22. Raphael BP, Nurko S, Jiang H, et al. Cisapride improves enteral tolerance in pediatric short-bowel syndrome with dysmotility. *J Pediatr Gastroenterol Nutr.* 2011;52(5):590-594.

23. Wymer KM, Anderson BB, Wilkens AA, Gundeti MS. Megacystis microcolon intestinal hypoperistalsis syndrome: Case series and updated review of the literature with an emphasis on urologic management. *J Pediatr Surg.* 2016;51(9):1565-1573.

24. Feudtner C, Zhong W, Faerber J, Dai D, Feinstein J. Dying In America: Improving Quality and Honoring Individual Preferences Near the End of Life. March 19 2015; Appendix F, Pediatric End-of-Life and Palliative Care: Epidemiology and Health Service Use. Available at: <https://www.ncbi.nlm.nih.gov/books/NBK285690/>.
